# Efficacy and Safety of JAK Inhibitors for Rheumatoid Arthritis: A Meta-Analysis

**DOI:** 10.3390/jcm11154459

**Published:** 2022-07-30

**Authors:** Faping Wang, Xiaoju Tang, Min Zhu, Hui Mao, Huajing Wan, Fengming Luo

**Affiliations:** 1 Laboratory of Pulmonary Immunology and Inflammation, Department of Respiratory and Critical Care Medicine, Frontiers Science Center for Disease-Related Molecular Network, West China Hospital, Sichuan University, Chengdu 610041, China; wangfpscu@gmail.com (F.W.); tangxiaoju888@hotmail.com (X.T.); huaxizhumin@wchscu.cn (M.Z.); merrmh@126.com (H.M.); 2 Clinical Research Center for Respiratory Disease, West China Hospital, Sichuan University, Chengdu 610041, China

**Keywords:** rheumatoid arthritis, JAK, inhibitors, meta-analysis, systematic review

## Abstract

Background: More and more trials have been conducted. We aimed to assess the efficacy and safety of different JAKinibs in RA. Methods: A systematic search of randomized controlled trials (RCTs) with JAKinib treatment in RA published in the Medline, Embase, and Cochrane databases up to May 2021 was performed. Results: 37 trials involving 15,174 patients were identified. Pooled analysis revealed that JAKinibs were associated with significant therapeutic improvement in RA patients as determined by ACR20 (RR = 2.03, 95% CI: 1.85 to 2.28) and HAQ-DI (MD = −0.31, 95% CI: −0.33 to −0.28) over placebo. Compared to placebo, JAKinib treatment was also associated with more adverse events (RR = 1.10, *p* < 0.001; RR = 1.29, *p* < 0.001; RR = 1.59, *p* = 0.02). Baricitinib and upadacitinib were related to more frequent adverse events (RR = 1.10; 95% CI: 1.01, 1.21; RR = 1.19; 95% CI: 1.11, 1.28) and infection (RR = 1.22; 95% CI: 1.09, 1.37; RR = 1.38; 95% CI: 1.22, 1.56), whereas only baricitinib was associated with more herpes zoster (RR = 3.15; 95% CI: 1.19, 8.33). Conclusions: JAKinibs were superior to placebo for improving signs, symptoms, and health-related quality of life in RA patients at short term, whereas the overall risk of adverse events and infections were greater with baricitinib and upadacitinib, and a higher risk of herpes zoster was only associated with baricitinib. More trials are needed to investigate the long-term safety.

## 1. Introduction

Rheumatoid arthritis (RA) is the most common autoimmune inflammatory arthritis in adults, which is characterized by chronic synovial joint inflammation, driven by immune system dysregulation [1]. The disease has a negative effect on quality of life and imposes a substantial economic burden on patients and society [2,3].

The cornerstone of RA treatment is conventional disease-modifying drugs (csDMARDs), like methotrexate (MTX). Over the last few decades, the evolving therapeutic landscape, like monoclonal antibodies or soluble receptors blocking pro-inflammatory cytokines such as TNF or IL-6 for RA, has seen major breakthroughs. However, many biological therapies are routinely administered in combination with nonbiological DMARDs, especially methotrexate. Although the outcome for patients with RA has improved in recent years, only approximately half of patients meet the criteria for low disease activity (≤3.2 on the on a 28-joint disease activity score using the erythrocyte sedimentation rate DAS28-4 (ESR)) or remission (<2.6 on the DAS28-4 (ESR)) [4]. In addition, their own side-effect profiles limited their use in patients [5]. Therefore, the development of orally available small molecules that inhibit intracellular signaling of cytokines and growth factors is an unmet need.

Janus kinases (JAKs) are a family of non-receptor tyrosine kinases linked to the intra-cellular domain of many cytokine receptors [6]. JAK phosphorylates cytokine-bound receptors, which triggers the intra-cellular molecular signaling that eventually modulates expression of genes involved in inflammation and tissue remodeling [6,7,8]. Studies have demonstrated that continuous activation of JAK/signal transduction and activation of transcription (STAT) signaling in RA synovial joints could induce a high level of matrix metalloproteinase gene expression, apoptosis of chondrocytes, and most prominently, apoptosis resistance of inflammatory cells in the synovial tissue, supporting that therapeutics targeting the JAK pathway may provide symptomatic relief for RA [9].

To date, a new field of clinical trials has been investigating the blockade of JAKkinase activity for the treatment of RA. Four isoforms of JAK were identified, including JAK1, JAK2, JAK3, and TYK2. Several JAK inhibitors (JAKinibs) with differing degrees of specificity for JAKs are in clinical trial. Tofacitinib is considered a pan-JAKinib, which mainly inhibits JAK1 and JAK3. Baricitinib is selective for JAK1 and JAK2, and peficitinib for JAK1 and JAK3. Filgotinib and upadacitinib are JAK1-selective agents, whereas decernotinib is a selective JAK3 inhibitor [1,5,10]. Now, tofacitinib, baricitinib, and upadacitinib have recently been approved by the FDA for the treatment of RA [11,12,13]. However, differences in efficacy and safety were seen, and which drug is relatively safe and effective is unclear. To help inform this debate, we conducted a systematic review and meta-analysis of all placebo-controlled randomized trials evaluating JAKinibs for RA to determine their pooled efficacy and safety relative to placebo.

## 2. Methods

### 2.1. Literature Searches and Study Selection

PubMed, Embase, and the Cochrane CENTRAL Library were searched without language restriction from inception to 5 May 2021 using the search terms ‘‘tofacitinib’’ or “CP-690550” or “baricitinib” or “LY3009104” or “Olumiant” or “upadacitinib” or “decernotinib” or “VX-509” or “peficitinib” or “ASP015K” or “filgotinib” or “GLPG0634” or “JAK inhibitors” and “rheumatoid arthritis’.’ All of the studies identified were reviewed independently by three investigators (FW, XT and MZ). Discrepancies were resolved through consensus and consultation with a third reviewer (FML) if needed. An example of the search strategy used to identify relevant trials published in Embase is presented in Table S2.

### 2.2. Inclusion and Exclusion Criteria

Eligible clinical trials were as follows: (1) adult patients with a diagnosis of RA and treated with JAKinibs; (2) double-blind, randomized, placebo-controlled studies; and (3) outcomes including the American College of Rheumatology 20% (ACR 20), ACR50, ACR70, Health Assessment Questionnaire—Disability Index (HAQ-DI, in which scores range from 0 to 3, with higher scores indicating greater disability), and adverse events. Studies presenting duplicate data or no safety data were excluded. No restrictions were applied to the length of follow-up and language.

### 2.3. Data Extraction and Outcome Measures

Data extraction was performed in duplicate by two independent reviewers (FW, XT, and MZ) using a standardized electronic data collection form. The following variables were extracted: authors, year of publication, study type, name of the study, clinicaltrials.gov number, doses used, number of patients, duration of study periods, and outcome measures. The ACR20 response was defined as at least 20% improvement in both the tender joint count and the swollen joint count and at least 20% improvement in three of five other core set measures: patient’s assessment of pain, patient’s global assessment of disease activity, physician’s global assessment of disease activity, patient’s assessment of physical function, or acute-phase reactant value [14]. The proportion of patients experiencing any adverse events (AE), serious AE (SAE), infections, and serious infections were extracted. Additionally, we captured the number of patients with herpes zoster (HZ), upper respiratory tract infections, thromboembolic events, MACE (major adverse cardiovascular events), and neoplasms.

### 2.4. Statistical Analyses

We calculated mean differences (MD) and risk ratio (RR). Study-level RRs or MDs with 95% confidence intervals (CI) were calculated in accordance with the intention-to-treat principle. Fixed-effects models were used when heterogeneity between studies was non-significant, and random-effects were used for analyses with significant heterogeneity. A *p*-value of less than 0.05 was considered statistically significant. For dose-ranging studies, data from all treatment doses were pooled. Heterogeneity was quantified using *I*^2^ (range, 0% to 100%; >50% indicates evidence of heterogeneity) [15,16]. In addition, the quality of the included trials and the risk of bias were assessed by using elements included in the Cochrane collaboration tool for assessing risk of bias. The funnel blot was determined and was used to evaluate the publication bias in our meta-analysis [17,18]. In addition, event rates for ACR20, ACR50, ACR70, AEs, SAEs, infections, serious infections, HZ, upper respiratory tract infections, thromboembolic events, MACE, and neoplasm among all studied outcomes were calculated and the numbers needed to treat (NNTs) or the numbers needed to harm (NNH). The NNT was equal to 1/|risk difference| according to Cochrane Handbook for Systematic Reviews of Interventions. Review Manager (RevMan version 5.3; The Cochrane Collaboration, n, Oxford, UK) was used for statistical analysis. The Grading of Recommendations Assessment, Development and Evaluation’s (GRADE’s) official GRADEpro software tool www.gradepro.org (accessed on 5 May 2021) was used to evaluate the certainty of evidence.

## 3. Results

### 3.1. Study Characteristics

**Figure 1:**
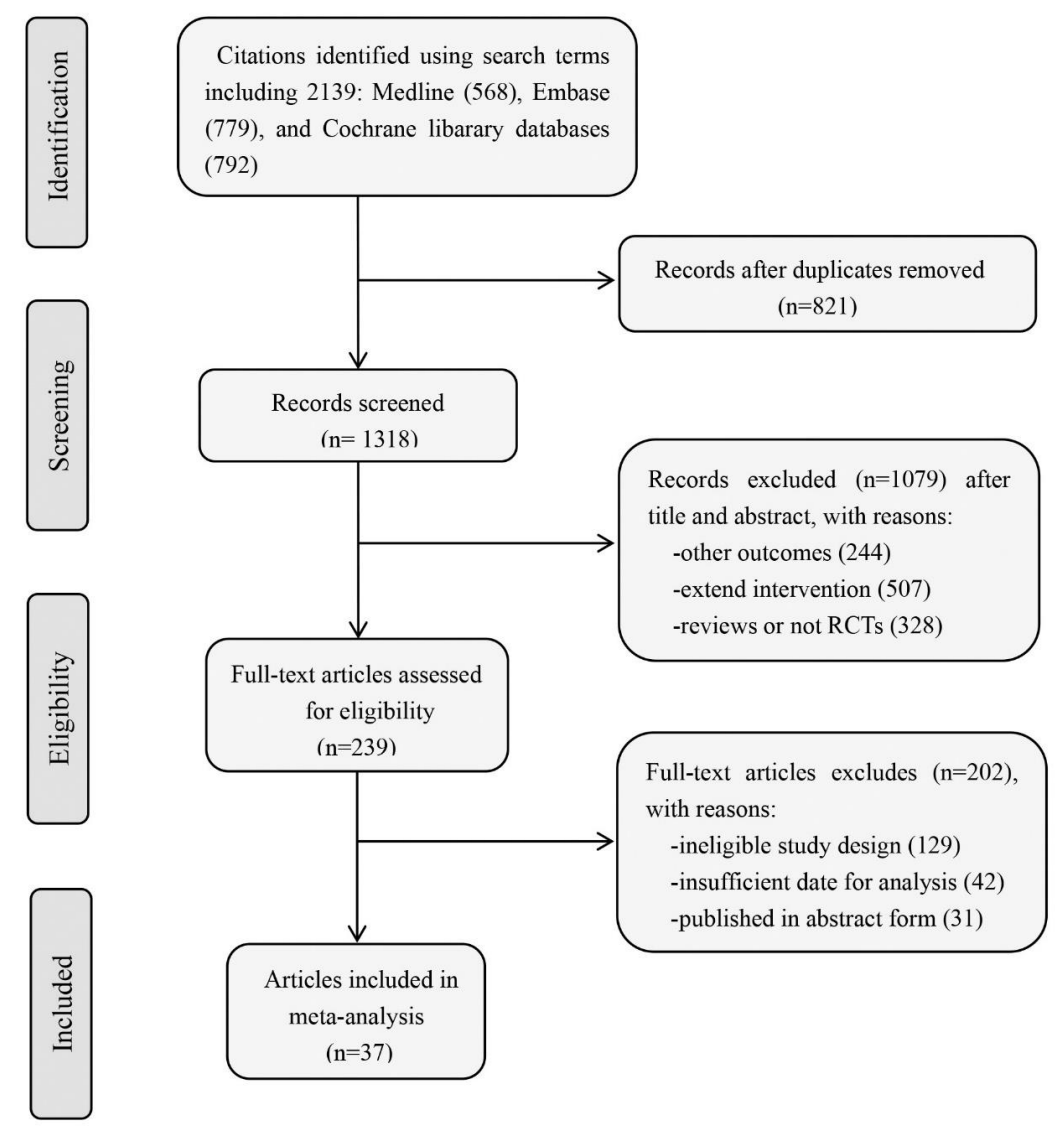
Flow chart of study selection procedure.

A total of 2139 manuscripts were identified (Figure 1): 568 from Medline, 779 from Embase, and 792 from The Cochrane Library. After removal of duplicates, we evaluated 1318 studies, of which 1079 were excluded based on title and abstract review. A full text assessment of the remaining 239 records was conducted. Finally, 36 studies (37 trials in total) carried out in different countries and on different ethnic backgrounds were included in this meta-analysis [19,20,21,22,23,24,25,26,27,28,29,30,31,32,33,34,35,36,37,38,39,40,41,42,43,44,45,46,47,48,49,50,51,52,53,54]. Seven trials were conducted in only one country, whereas the rest were performed in multiple countries.

A total of 37 RCTs and 15,174 participants in total were enrolled in this systematic review and meta-analysis, including 12 tofacitinib, 6 baricitinib, 6 upadacitinib, 3 decernotinib, 5 peficitinib, and 5 filgotinib. The baseline patient characteristics of trials are shown in Table 1. Duration of treatment ranged from 4 to 24 weeks. The characteristics of the included trials are summarized in Table 1. Key findings are summarized in Table 2.

### 3.2. Risk of Bias Assessment

**Figure 2:**
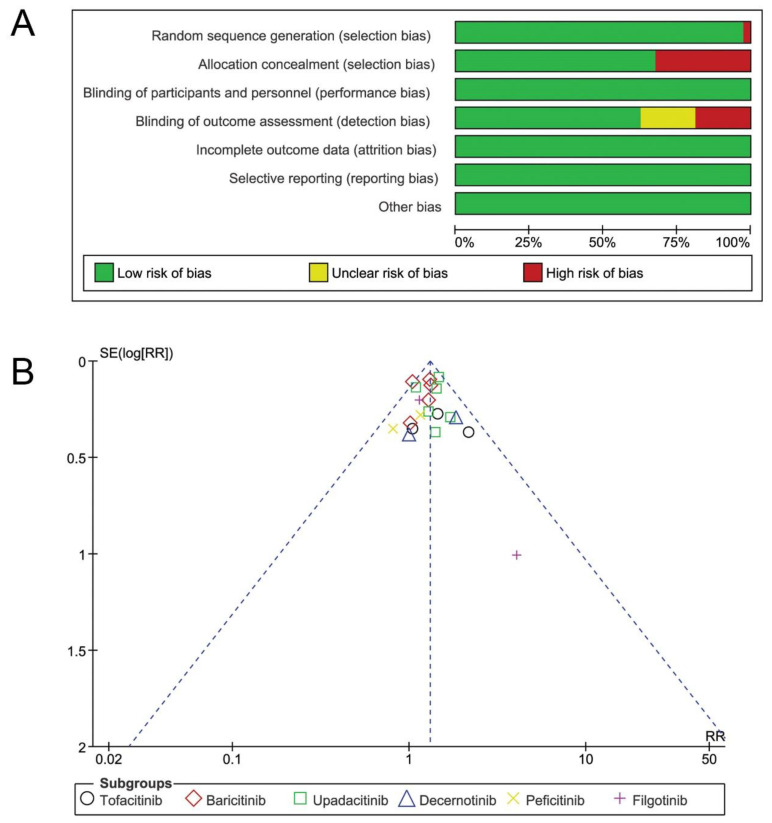
The risk of bias assessment and publication bias. (**A**) Risk of bias summary of included trials; (**B**) funnel plot of the included trials evaluating the effect of JAKinibs on adverse events. RR = relative risks.

All the studies included in the meta-analysis were deemed to be a low risk of bias (Figure 2A). Most studies used random sequence generation and allocation concealment. Blinding of study subjects and investigators was universally maintained by the use of placebo. All trials reported the outcome data; baselines of the subjects involved in the studies were similar. No evidence for publication bias was detected using the funnel plot (Figure 2B).

### 3.3. Efficacy

#### 3.3.1. ACR20, ACR50, and ACR70

All the studies reported the data of ACR20 except one [30]. The pooled effect of JAKinibs on ACR20 was significant (RR = 2.03, 95% CI: 1.85 to 2.23, *p* < 0.001, NNT = 4), with moderate heterogeneity (*I*^2^ = 65%, *p* < 0.001) (Figure S1). Figure S1 shows that ACR20 response was higher for decernotinib than other JAKinibs (RR = 2.61, 95% CI: 1.70 to 4.01, *p* < 0.001), with minimal heterogeneity (*I*^2^ = 31%), but the results should be interpreted with caution due to the small number of studies involved. Filgotinib seemed to be the least effective drug in terms of ACR20 (RR = 1.80, 95% CI: 1.43 to 2.27, *p* < 0.001). Certainty in the evidence was judged to be moderate, mainly because of the possibility of publication bias (Table 3). Figures S2 and S3 showed that JAKinibs were more effective than placebo on ACR50 (RR = 3.12, 95% CI: 2.48 to 3.83, *p* < 0.001, NNT = 5) and ACR 70 (RR = 3.87, 95% CI: 3.02 to 4.97, *p* < 0.001, NNT = 7), with significant heterogeneity (*I*^2^ = 84% and *I*^2^ = 56%, respectively). Sensitivity analysis indicated that varied subjects among studies may contribute to the heterogeneity of ACR20 and ACR50 (Table S1).

#### 3.3.2. HAQ-ID

Nineteen trials totaling 8703 subjects were included. Peficitinib was evaluated in only one study. Overall, JAKinib administration produced a significant decrease in HAQ-ID (MD= −0.31, 95% CI: −0.34 to −0.28, *p* < 0.001) compared to placebo (Figure S4). There was no significant heterogeneity among the included studies (*I*^2^ = 0%, *p* = 0.69). Among the subgroups, tofacitinib seemed to show the most beneficial effect on HAQ-ID (MD = −0.34, 95% CI: −0.39 to −0.28, *p* < 0.001), without significant heterogeneity (*I*^2^ = 0%, *p* = 0.94).

### 3.4. Safety

#### 3.4.1. AEs and SAEs

Across all studies, 7897 of 14,260 randomized patients experienced one or more AEs. The pooled RR was 1.10 (95% CI: 1.05–1.14, NNT = 30), which shows that the highest AE incidence was slightly in the JAKinib group (*p* < 0.001), with mild heterogeneity (*I*^2^ = 25%, *p* = 0.09) (Figure S5). Upadacitinib seemed to show the highest trend towards increasing in any adverse events (RR = 1.19, 95% CI, 1.11–1.28, *p* < 0.001, *I*^2^ = 7%) compared to placebo. On subgroup analysis, tofacitinib, decernotinib, peficitinib, and filgotinib seemed to show similar AEs to the placebo group (RR = 1.06, 1.32, 1.04, 0.96 *p* = 0.16, 0.07, 0.41, and 0.57 respectively). The GRADE quality of adverse events was judged to be high (Table 3), and the absolute effect was 53 fewer per 1000 (from 27 fewer to 74 more). A total of 34 studies evaluated SAEs, with a pooled RR of 0.94 (95% CI, 0.77–1.15, *I*^2^ = 0%, NNT = 1000) (Figure S6), and subgroup analysis showed that none of the JAKinibs were associated with a trend of high SAE. Certainty in the evidence about the risks of serious adverse events was judged as moderate (Table 3).

#### 3.4.2. Infections and Serious Infections

There were 21 studies that evaluated infections, and treatment with JAKinibs was associated with a significantly increased risk of infections (RR = 1.29, 95% CI, 1.19–1.39, *p* < 0.001, *I*^2^ = 0%, NNT = 30) (Figure S7). Tofacitinib, decernotinib, peficitinib, and filgotinib were not associated with high incidence of infections (RR = 1.39, 1.43, 1.01, 1.50, *p* = 0.05, 0.23, 0.96, and 0.44, respectively), but only a small number of trials were analyzed for each. Certainty in the evidence about the risk of infections was high. Serious infections occurred in a similar proportion of patients in the placebo and JAKinib groups without heterogeneity (RR = 1.30, 95% CI, 0.92–1.86, *p* = 0.14, *I*^2^ = 0%, NNT = 143) (Figure S8). Proportions of patients with serious infections were similar across all subgroups. Certainty in the evidence about the risk of serious infections was high (Table 3).

#### 3.4.3. HZ

A total of 25 studies reported HZ. Prominent risk of HZ was observed in the JAKinib group compared to placebo (RR = 1.59, 95% CI, 1.09–2.32, *p* = 0.02, NNT = 77). Heterogeneity was not statistically significant (*I*^2^ = 0%, *p* = 0.79) (Figure S9). However, HZ risk was higher only for baricitinib and not other JAkinibs (RR = 3.15, 95% CI, 1.19–8.33, *p* = 0.02, *I*^2^ = 0%). However, the pooled effect of JAKinibs on HZ was not significant, and baricitinib groups were excluded (RR = 1.41; 95% CI: 0.94–2.11, *p* = 0.10), which indicates that the baricitinib groups significantly affected the pooled results. Certainty in the evidence about the risk of herpes zoster was high (Table 3).

#### 3.4.4. Upper Respiratory Tract Infections

Fifteen trials were included in the analysis. Overall, JAKinibs showed no significant increase in risk of upper respiratory tract infections compared with placebo (RR = 1.26, 95% CI, 0.97–1.63, *p* = 0.08, *I*^2^ = 0%, NNT = 72) (Figure S10). In addition, all of these drugs resulted in a numerically but not statistically increased risk of upper respiratory tract infections (RR = 1.20, 1.22, 1.34, 1.24, 1.60, 0.89, *p* = 0.52, 0.38, 0.44, 0.78, 0.27, and 0.22, respectively). The certainty in the evidence was moderate (Table 3).

#### 3.4.5. Thromboembolic Events

Only 13 trials reported thromboembolic events, and the pooled results of JAKinibs revealed no significant increased risk compared to placebo (RR = 1.04, 95% CI, 0.38–2.84, *p* = 0.94, *I*^2^ = 0%, NNT = 500) (Figure S11). Unfortunately, only a few trials reported the data of this outcome, and certainty in the evidence was very low due to the wide confidence intervals and suspected publication bias (Table 3).

#### 3.4.6. MACE

Sixteen trials reported the MACE, and the pooled results of JAKinibs revealed no significant increased risk compared to placebo (RR = 1.02, 95% CI, 0.45–2.34, *p* = 0.96, *I*^2^ = 0%, NNT = 500) (Figure S12). Less than half of the trials reported the data of this outcome, and certainty in the evidence was very low due to the wide confidence intervals and suspected publication bias (Table 3).

#### 3.4.7. Neoplasm

Nineteen trials were included in the analysis, and the pooled results of JAKinibs revealed no significant increased risk compared to placebo (RR = 1.70, 95% CI, 0.74–3.89, *p* = 0.96, *I*^2^ = 0%, NNT = 250) (Figure S13). Some trials did not provide the data of this outcome, and certainty in the evidence was very low due to the wide confidence intervals and suspected publication bias (Table 3).

## 4. Discussion

This meta-analysis investigated the efficacy and safety of six different oral JAKinibs in the treatment of patients with RA. All JAKinibs were found to be consistently more effective than placebo. However, the safety issues should be considered with caution. Overall, JAKinibs increased the adverse events, risk of infection, and herpes zoster compared to placebo. Subgroup analysis revealed that baricitinib was the only JAKinib to show significantly higher risk of herpes zoster. Additionally, baricitinib and upadacitinib significantly increased the adverse events and infections compared to placebo.

RA is a chronic autoimmune disease characterized by systemic, destructive, and progressive inflammatory polyarthritis, driven by immune system dysregulation [14]. JAK/STAT signaling pathway is involved in the pathogenesis of inflammatory and autoimmune diseases such as RA, psoriasis, and inflammatory bowel disease [7]. Given the major role played by JAKs and STATs in the pathogenesis of autoimmunity [55,56], small molecules targeted against JAKs or JAKinibs are developed. However, only 5 mg tofacitinib taken twice daily, 2 mg baricitinib taken daily, and 15 mg upadacitinib taken daily are FDA-approved doses for the treatment of adult patients with moderately to severely active RA with a prior inadequate response or intolerance to methotrexate [57,58,59]. Since there were no head-to-head randomized trials to compare different JAKinibs, the evidence is inadequate for drawing robust conclusions of the benefit–risk for each JAKinib.

Previous meta-analysis [60] and network meta-analysis [61] also evaluated JAKinibs, but they only included tofacitinib, baricitinib, and upadacitinib. Consistent with the previous meta-analysis [60], a statistically significant increased risk of HZ was apparent with baricitinib. Futhermore, this study also demonstrated a notable increased risk of infections with baricitinib and upadacitinib, which was not observed in the previous meta-analysis. This is attributed to more trials of upadacitinib, which were included in this study. Consistent with the previous network meta-analysis, a notable increased risk of SAE with JAKinibs was not observed; however, that network analysis did not include AE analysis in the report and included fewer patients compared to the current study, whereas a significant increased risk of AE was observed in our study.

Based on the pooled analyses, JAKinibs could show a significant benefit in achieving ACR20 responses compared to placebo. Although decemotinib seemed to be the most effective drug followed by tofacitinib among all the JAKinibs according to the results, we had no confidence in this due to the small number of trials and patients (only three trials and 316 patients involved), as well as the relatively short duration of the trials (the longest follow-up time was 24 weeks). Anyway, these six JAKinibs showed no huge efficacy differences in terms of ACR20. With regards to HAQ-ID, the results showed that treatment with JAKinibs led to a statistically significant improvement from baseline compared to placebo. The minimal clinically important difference in HAQ-DI was defined as 0.22 or more [62,63]. Importantly, all the improvements caused by JAKinibs were higher than 0.22. Tofacitinib demonstrated the most effective benefit in HAQ-DI, followed by filgotinib, but the results of filgotinib need to be interpreted with caution, as only three trials were included.

For safety, baricitinib and upadacitinib seemed to be only two JAKinibs that could increase the risk of AEs, infections, and HZ compared to placebo. However, the results of decemotinib, peficitinib, and filgotinib are limited (less than five trials included for each); we are not confident about the results. Additionally, the short duration of the trials related to these three JAKinibs limits any conclusions that can be made on the safety of longer-term use. Therefore, more data are needed to support the safety profile of decemotinib, peficitinib, and filgotinib. Considering that large phase 3 trials of filgotinib, decernotinib, and peficitinib are still ongoing, we recognize that the small number of patients treated for a short period of time was insufficient to reach maximal efficacy levels or to obtain a full safety picture of them. Therefore, the results related to these three JAKinibs should be interpreted with caution. Of note, consistent with previous meta-analysis [64,65], baricitinib was found to increase the risk of HZ. However, the pathogenesis underlying the risk of HZ is poorly understood. The potential mechanisms explaining this association may have to do with the role of JAK2, because baricitinib is a more highly selective inhibitor of JAK2 than other JAKinibs. Besides, Japanese and Korean populations appeared to be more likely to suffer from HZ infections [1]. HZ may be significantly influenced by ethnicity and geographical differences, according to different studies. 

Overall, tofacitinib, decemotinib, peficitinib, and filgotinib are superior to baricitinib and upadacitinib regarding the safety profile. Considering the low confidence for results of decemotinib, peficitinib, and filgotinib, tofacitinib seemed to the most beneficial and safe JAKinib comparing to baricitinib and upadacitinib (more AE, infections, and HZ occur). However, the FDA and post-marketing safety surveillance have identified a higher risk of pulmonary embolism and death with the 10 mg twice daily dose of tofacitinib in RA patients [66]. Although this meta-analysis provided no support of thromboembolic events warning across all the JAKinibs, this analysis could not be extended to the real world due to lack of data. On the contrary, a real-world data analysis revealed similar incidence rates of thromboembolic events across tofacitinib doses [67]. Venous thromboembolic events, including pulmonary embolism, have also emerged for both baricitinib and upadacitinib [68]. A recent meta-analysis evaluated the venous thromboembolism risk of JAKinibs in immune-mediated inflammatory diseases; however, their results did not provide evidence of an increased risk for JAKinibs [69]. In addition, whether the increased thromboembolic risk is related to RA disease activity and drug safety is uncertain. Thus, current information regarding this risk is not confirmed yet and further accruing, full details of thromboembolic events in trials of JAKinibs need to be published.

Several limitations deserve consideration. First, the varied severity and baseline therapy of RA among studies limited generalizability to individual patients. Second, there were limited trials for the effect of decernotinib, peficitinib, and fligotinib. Third, a significant heterogeneity was noted among trials evaluating ACR 20, ACR50, and ACR70. Although a random-effects model was used, the correction is only partial, and possible sources of heterogeneity might include ethnicity and geographic factors, different enrollment criteria of participants, and definable differences in study populations included. Fourth, in some trials, a subgroup or all the placebo patients switched to treatment groups to address ethical concerns about continuing placebo in patients with active disease; therefore, only short-term data for comparing treatment with placebo were included, which prevented us from analyzing the long-term adverse effects of JAKinibs.

## 5. Conclusions

In conclusion, in this systematic review and meta-analysis, we demonstrate that JAKinibs are effective at reducing RA signs and symptoms of RA, and improve health-related quality of life, but the safety concerns should be paid attention. Increased risk of infections and AE were observed in baricitinib and upadacitinib, whereas only baricitinib statistically increased the risk of HZ. However, this study was limited by its short duration (less than 24 weeks). Further trials are necessary to assess long-term safety, especially for decernotinib, peficitinib, and fligotinib.

## Figures and Tables

**Table 1:** Characteristics of included trials. Only data including placebo and the doses of interest are selected; therefore, the selected study duration is different from the original research.

Author (Year)	Region	Trial Identifier	Follow-Up	No. of Patients	Dose
**Tofacitinib**					
Kremer 2009 [19]	Worldwide	NCT00147498	6 weeks	264	5, 15, 30 mg twice daily
Tanaka 2011 [20]	Japan	NCT00603512	12 weeks	140	1, 3, 5, 10, 15 mg twice daily
Vollenhoven 2012 [21]	America and Europe	NCT00853385	24 weeks	513	5, 10 mg twice daily
Fleischmann 2012a [22]	Worldwide	NCT00550446	24 weeks	274	1, 3, 5, 10, 15 mg twice daily
Fleischmann 2012 b [23]	Worldwide	NCT00814307	24 weeks	611	5, 10 mg twice daily
Kremer 2012 [24]	America and Europe	NCT00413660	12 weeks	507	1, 3, 5, 10, 15 mg twice daily, 20 mg/day
Burmester 2013 [25]	America and Europe	NCT00960440	12 weeks	399	5, 10 mg twice daily
Kremer 2013 [26]	Worldwide	NCT00856544	24 weeks	792	5, 10 mg twice daily
Heijde 2013 [27]	Worldwide	NCT00847613	24 weeks	797	5, 10 mg twice daily
Boyle 2015 [28]	Worldwide	NCT00976599	4 weeks	29	10 mg twice daily
Tanaka 2015 [29]	Japan	NCT00687193	12 weeks	317	1, 3, 5, 10, 15 mg twice daily, 20 mg/day
Kremer 2015 [30]	Worldwide	NCT01484561	6 weeks	148	10 mg twice daily
**Baricitinib**					
Keystone 2015 [31]	Worldwide	NCT01185353	12 weeks	301	1, 2, 4, 8 mg once daily
Tanaka 2016 [32]	Japan	NCT01469013	12 weeks	145	1, 2, 4, 8 mg once daily
Genovese 2016 [33]	Worldwide	NCT01721044	24 weeks	527	2, 4 mg once daily
Taylor 2017 [34]	Worldwide	NCT01710358	24 weeks	1307	4 mg once daily
Dougados 2017 [35]	Worldwide	NCT01721057	24 weeks	684	2, 4 mg once daily
Li 2020 [36]	China, Brazil, Argentina	NCT02265705	12 weeks	290	4 mg once daily
**Upadacitinib**					
Kremer 2016 [37]	Worldwide	NCT01960855	12 weeks	276	3, 6, 12, 18 mg twice daily
Genovese 2016 [38]	Worldwide	NCT02066389	12 weeks	299	3, 6, 12, 18 mg twice daily, 24 mg once daily
Burmester 2018 [39]	Worldwide	NCT02675426	12 weeks	661	15, 30 mg once daily
Genovese 2018 [40]	Worldwide	NCT02706847	24 weeks	499	15, 30 mg once daily
Fleischmann 2019 [41]	Worldwide	NCT02629159	12 weeks	1304	15 mg once daily
Kameda 2020 [42]	Japan	NCT02720523	12 weeks	148	15, 30 mg once daily
**Decernotinib**					
Fleischmann 2015 [43]	Worldwide	NCT01052194	12 weeks	204	25, 50, 100, 150 mg twice daily
Genovese 2016a [44]	Worldwide	NCT01754935	12 weeks	43	100, 200, 300 mg once daily
Genovese 2016b [45]	Worldwide	NCT2011-004419-22	24 weeks	358	100, 150, 200 mg once daily, 100 mg twice daily
**Peficitinib**					
Takeuchi 2016 [46]	Japan	NCT01649999	12 weeks	281	25, 50, 100, 150 mg once daily
Genovese 2017 [47]	Worldwide	NCT01565655	12 weeks	289	25, 50, 100, 150 mg once daily
Kivitz 2017 [48]	Worldwide	NCT01554696	12 weeks	378	25, 50, 100, 150 mg once daily
Takeuchi 2019 [49]	Japan	NCT02305849	12 weeks	519	100, 150 mg once daily
Tanaka 2019 [50]	Japan, Korea, Taiwan	NCT02308163	12 weeks	307	100, 150 mg once daily
**Filgotinib**					
Kavanaugh 2017 [51]	Worldwide	NCT01894516	24 weeks	283	50, 100, 200 mg once daily
Westhovens 2017 [52]	Worldwide	NCT01888874	24 weeks	594	50, 100, 200 mg once daily and twice daily
Vanhoutte 2017 1 [53]	Republic of Moldova	NCT01384422	4 weeks	36	100 mg twice daily or 200 once daily
Vanhoutte 2017 2 [53]	Worldwide	NCT01668641	4 weeks	91	30, 75, 150, 300 mg once daily
Genovese 2019 [54]	Worldwide	NCT02873936	24 weeks	449	100, 200 mg once daily

Worldwide: more than three countries. Only data including placebo are selected; therefore, the selected study duration is different from the original research.

**Table 2:** Summary of results stratified by JAKinibs compared to placebo corresponding to respective outcomes.

Outcomes	Studies (*n*)	RR	Lower 95% CI	Upper 95% CI	*I* ^2^	Outcomes	Studies (*n*)	RR	Lower 95% CI	Upper 95% CI	*I* ^2^
**ACR-20**						**Infections**					
All RCTs	36	2.03	1.85	2.23	65%	All RCTs	21	1.29	1.19	1.39	0%
Tofacitinib	11	2.21	1.86	2.63	52%	Tofacitinib	3	1.30	1.00	1.94	0%
Baricitinib	6	1.95	1.57	2.42	78%	Baricitinib	5	1.22	1.09	1.37	0%
Upadacitinib	6	1.99	1.68	2.36	64%	Upadacitinib	6	1.38	1.22	1.56	0%
Decernotinib	3	2.61	1.70	4.01	31%	Decernotinib	2	1.43	0.80	2.58	37%
Peficitinib	5	2.01	1.32	3.05	84%	Peficitinib	2	1.01	0.66	1.56	0%
Filgotinib	5	1.80	1.43	2.27	46%	Filgotinib	2	1.50	0.53	4.20	37%
**ACR-50**						**ACR-70**					
All RCTs	35	3.12	2.48	3.93	84%	All RCTs	33	3.87	3.02	4.97	56%
Tofacitinib	11	3.43	2.30	5.12	78%	Tofacitinib	11	4.15	2.21	7.80	74%
Baricitinib	6	2.73	2.03	3.66	64%	Baricitinib	6	3.81	2.97	4.89	0%
Upadacitinib	6	2.25	1.12	4.52	96%	Upadacitinib	6	4.53	3.53	5.83	0%
Decernotinib	3	4.72	2.48	8.96	0%	Decernotinib	3	4.06	1.50	10.98	0%
Peficitinib	5	2.84	1.42	5.70	82%	Peficitinib	5	3.64	1.32	10.05	73%
Filgotinib	4	5.56	2.79	11.06	11%	Filgotinib	2	3.41	0.94	12.40	45%
**HAQ-DI**						**Serious infections**					
All RCTs	20	−0.31	−0.34	−0.28	0%	All RCTs	29	1.30	0.92	1.86	0%
Tofacitinib	7	−0.34	−0.39	−0.28	0%	Tofacitinib	8	1.35	0.72	2.55	0%
Baricitinib	2	−0.24	−0.33	−0.15	0%	Baricitinib	6	0.91	0.48	1.71	0%
Upadacitinib	5	−0.31	−0.36	−0.26	0%	Upadacitinib	6	1.92	0.83	4.47	4%
Decernotinib	2	−0.24	−0.48	−0.01	72%	Decernotinib	2	2.58	0.49	13.63	0%
Peficitinib	1	−0.22	−0.42	−0.02	-	Peficitinib	4	2.63	0.59	11.73	0%
Filgotinib	3	−0.33	−0.44	−0.22	44%	Filgotinib	3	0.67	0.18	2.44	0%
**Adverse events**						**Herpes zoster**					
All RCTs	34	1.10	1.05	1.14	25%	All RCTs	25	1.59	1.09	2.32	0%
Tofacitinib	11	1.06	0.98	1.15	29%	Tofacitinib	4	1.28	0.72	2.29	0%
Baricitinib	5	1.10	1.01	1.21	48%	Baricitinib	6	3.15	1.19	8.33	0%
Upadacitinib	6	1.19	1.11	1.28	7%	Upadacitinib	6	1.25	0.56	2.81	0%
Decernotinib	3	1.32	0.97	1.78	40%	Decernotinib	1	1.79	0.09	34.04	-
Peficitinib	5	1.04	0.94	1.16	0%	Peficitinib	5	2.13	0.51	8.92	37%
Filgotinib	5	0.96	0.84	1.10	0%	Filgotinib	3	0.97	0.21	4.51	0%
**Serious adverse events**						**Upper respiratory infection**					
All RCTs	34	0.94	0.77	1.15	0%	All RCTs	15	1.26	0.97	1.63	0%
Tofacitinib	11	0.74	0.47	1.18	20%	Tofacitinib	8	1.20	0.69	2.10	33%
Baricitinib	6	0.92	0.65	1.31	0%	Baricitinib	2	1.22	0.78	1.89	0%
Upadacitinib	6	1.72	0.92	3.25	18%	Upadacitinib	1	1.34	0.63	2.83	-
Decernotinib	3	1.47	0.58	3.71	0%	Decernotinib	1	1.24	0.28	5.52	-
Peficitinib	5	0.95	0.46	1.96	0%	Peficitinib	2	1.60	0.69	3.67	0%
Filgotinib	3	0.70	0.24	2.07	46%	Filgotinib	1	0.89	0.30	2.60	-
**Thromboembolic events**						**MACE**					
All RCTs	13	1.04	0.38	2.84	0%	All RCTs	16	1.02	0.45	2.34	0%
Tofacitinib	2	0.19	0.01	2.91	35%	Tofacitinib	3	2.43	0.31	19.07	0%
Baricitinib	2	2.38	0.27	20.84	0%	Baricitinib	5	0.59	0.10	3.40	21%
Upadacitinib	5	1.65	0.33	8.35	0%	Upadacitinib	5	1.17	0.32	4.22	0%
Decernotinib	1	0.77	0.03	18.52	-	Decernotinib	2	0.76	0.08	7.22	0%
Peficitinib *	2	-	-	-	-	Peficitinib *	1	-	-	-	-
Filgotinib	1	1.49	0.06	36.24	-	Filgotinib	0	-	-	-	-
Neoplasms											
All RCTs	19	1.70	0.74	3.89	0%						
Tofacitinib	1	9.50	0.56	162.20	-						
Baricitinib	5	1.03	0.26	4.10	0%						
Upadacitinib	6	1.50	0.40	5.54	0%						
Decernotinib	5	2.92	0.35	24.20	0%						
Peficitinib	-	-	-	-	-						
Filgotinib *	2	-	-	-	-						

* No events in placebo or JAKinib group. RR: risk ratio; CI: confidence intervals; RCT: randomized controlled trials; ACR-20: American College of Rheumatology 20%; ACR-50: American College of Rheumatology 50%; ACR-70: American College of Rheumatology 70%; HAQ-DI: Health Assessment Questionnaire—Disability Index; MACE: major adverse cardiovascular events.

**Table 3:** Summary of findings, including GRADE quality assessment of evidence from trials.

Variables	No. of Studies	No. of Patients		Effect		NNT/NNH	Quality of the Evidence (GRADE)	Quality Domains and Assessments	Importance
		JAKinibs Group	Placebo Group	Relative (95% CI)	Absolute (95% CI)				
ACR20	36	6191/10,361 (59.8%)	1251/4255 (29.4%)	RR 2.03 (1.85 to 2.23)	303 more per 1000 (from 250 more to 362 more)	4	⨁⨁⨁◯ MODERATE	Risk of bias: not serious Inconsistency: not serious Indirectness: not serious Imprecision: not serious Other: publication bias strongly suspected ^a^	Critical
ACR50	35	3800/10,061 (37.8%)	551/4107 (13.4%)	RR 3.10 (2.63 to 3.66)	282 more per 1000 (from 219 more to 357 more)	5	⨁⨁⨁◯ MODERATE	Risk of bias: not serious Inconsistency: not serious Indirectness: not serious Imprecision: not serious Other: publication bias strongly suspected ^a^	Important
ACR70	33	1946/9963 (19.5%)	212/4078 (5.2%)	RR 3.87 (3.02 to 4.97)	149 more per 1000 (from 105 more to 206 more)	7	⨁⨁⨁◯ MODERATE	Risk of bias: not serious Inconsistency: not serious Indirectness: not serious Imprecision: not serious Other: publication bias strongly suspected ^a^	Important
Adverse events	34	5735/10,181 (56.3%)	2162/4079 (53.0%)	RR 1.10 (1.05 to 1.14)	53 more per 1000 (from 27 more to 74 more)	30	⨁⨁⨁⨁ HIGH	Risk of bias: not serious Inconsistency: not serious Indirecteness: not serious Imprecision: not serious Other: none	Critical
Serious adverse events	34	321/9898 (3.2%)	136/4181 (3.3%)	RR 0.94 (0.77 to 1.15)	2 fewer per 1000 (from 7 fewer to 5 more)	1000	⨁⨁⨁◯ MODERATE	Risk of bias: not serious Inconsistency: seriousIndirectness: not serious Imprecision: not serious Other: publication bias strongly suspected ^a^	Important
Infection	21	1696/6292 (27.0%)	695/2948 (23.6%)	RR 1.29 (1.19 to 1.39)	68 more per 1000 (from 45 more to 92 more)	30	⨁⨁⨁⨁ HIGH	Risk of bias: not serious Inconsistency: not serious Indirectness: not serious Imprecision: not serious Other: none	Important
Serious infection	29	155/9043 (1.7%)	37/3879 (1.0%)	RR 1.30 (0.92 to 1.86)	3 more per 1000 (from 1 fewer to 8 more)	143	⨁⨁⨁⨁ HIGH	Risk of bias: not serious Inconsistency: serious Indirectness: not serious Imprecision: not serious Other: none	Important
Herpes zoster	25	160/7700 (2.1%)	28/3533 (0.8%)	RR 1.59 (1.09 to 2.32)	5 more per 1000 (from 1 more to 10 more)	77	⨁⨁⨁⨁ HIGH	Risk of bias: not serious Inconsistency: serious ^b^ Indirectness: not serious Imprecision: serious Other: none	Important
Upper respiratory infection	15	315/5491 (5.7%)	74/1733 (4.3%)	RR 1.26 (0.97 to 1.63)	11 more per 1000 (from 1 more to 27 more)	72	⨁⨁⨁◯ MODERATE	Risk of bias: not serious Inconsistency: not serious Indirectness: not serious Imprecision: not serious Other: publication bias strongly suspected ^a^	Not important
Thromboembolic events	13	12/4455 (0.3%)	3/2241 (0.1%)	RR 1.04 (0.38 to 2.84)	0 fewer per 1000 (from 1 fewer to 2 more)	500	⨁⨁◯◯ LOW	Risk of bias: not serious Inconsistency: not serious Indirectness: not serious Imprecision: serious ^b^: Other: publication bias strongly suspected ^a^	Important
MACE	16	20/5704 (0.4%)	5/2735 (0.2%)	RR 1.02 (0.45 to 2.34)	0 fewer per 1000 (from 1 fewer to 2 more)	500	⨁⨁◯◯ LOW	Risk of bias: serious Inconsistency: not serious Indirectness: not serious Imprecision: serious ^b^: Other: publication bias strongly suspected ^a^	Not IMPORTANT
Neoplasms	19	27/5885 (0.5%)	4/3051 (0.1%)	RR 1.70 (0.74 to 3.89)	1 fewer per 1000 (from 0 fewer to 4 more)	250	⨁⨁◯◯ LOW	Risk of bias: serious Inconsistency: not serious Indirectness: not serious Imprecision: serious ^b^: Other: publication bias strongly suspected ^a^	Not IMPORTANT

CI: confidence interval; RR: risk ratio; ^a^ publication bias, Egger’s *p* = 0.00; ^b^ wide confidence interval. NNT: number needed to treat; NNH: number needed to harm. MACE: major adverse cardiovascular events; ⨁: the certainty of evidence is high; ◯: the certainty of evidence is low

## Data Availability

Please see the original included articles.

## Supplementary Materials

The following supporting information can be downloaded at: www.mdpi.com/article/10.3390/jcm11154459/s1. Figure S1: Forest plot of the effect of JAKinibs on ACR20 (American College of Rheumatology 20 response rates) verse placebo. Figure S2: Forest plot of the effect of JAKinibs on ACR50 (American College of Rheumatology 50 response rates) verse placebo. Figure S3: Forest plot of the effect of JAKinibs on ACR70 (American College of Rheumatology 70 response rates) verse placebo. Figure S4: Forest plot of the effect of JAKinibs on HAQ-ID (Health Assessment Questionnaire–Disability Index) verse placebo. Figure S5: Forest plot of the effect of JAKinibs on AEs. Figure S6: Forest plot of the effect of JAKinibs on SAEs. Figure S7: Forest plot of the effect of JAKinibs on infections. Figure S8: Forest plot of the effect of JAKinibs on serious infections. Figure S9: Forest plot of the effect of JAKinibs on HZ. Figure S10: Forest plot of the effect of JAKinibs on upper respiratory infection. Figure S11: Forest plot of the effect of JAKinibs on thromboembolic events. Figure S12: Forest plot of the effect of JAKinibs on MACE (major adverse cardiovascular events). Figure S13: Forest plot of the effect of JAKinibs on neoplasms. Table S1: Sensitivity analyses of ACR20 and ACR50 in RCTs Stratification. Table S2: Search strategies. PRISMA 2009 Checklist [70].

## Abbreviations

RA	Rheumatoid arthritis
csDMARDs	Conventional disease-modifying-drugs
MTX	Methotrexate
DAS28-4[ESR]	28-joint disease activity score using erythrocyte sedimentation rate
JAK	Janus kinases
STAT	Signal transduction and activator of transcription
JAKinibs	JAK inhibitors
ACR 20	American College of Rheumatology 20%
HAQ-DI	Health Assessment Questionnaire—Disability Index
AE	Adverse events
SAE	Serious adverse events
HZ	Herpes zoster
MD	Mean differences
RR	Risk ratio
CI	Confidence intervals
