# Supplementary material for: Efficacy and Safety of JAK Inhibitors for Rheumatoid Arthritis: A Meta-Analysis
Source: J Clin Med. 2022 Jul 30;11(15):4459. doi: 10.3390/jcm11154459 (PMC9369647; doi:10.3390/jcm11154459)
Supplement: Supplementary file 1 [file jcm-11-04459-s001.zip › jcm-1724056-supplementary.pdf]

Supplemental material

Efficacy and safety of JAK inhibitors for Rheumatoid Arthritis: A meta-analysis

Faping Wang, MD[1,2#], Xiaoju Tang, MD[1,2], Min Zhu, MD[1,2], Hui Mao, MD[1,2], Huajing Wan PhD[1,2], Fengming Luo, MD[1,2*]*

Supplemental Figures

***Figure S1**: Forest plot of the effect of JAKinibs on ACR20 (American College of Rheumatology 20 response rates) verse placebo. Randomized-effects model. RR=Relative Risks.*

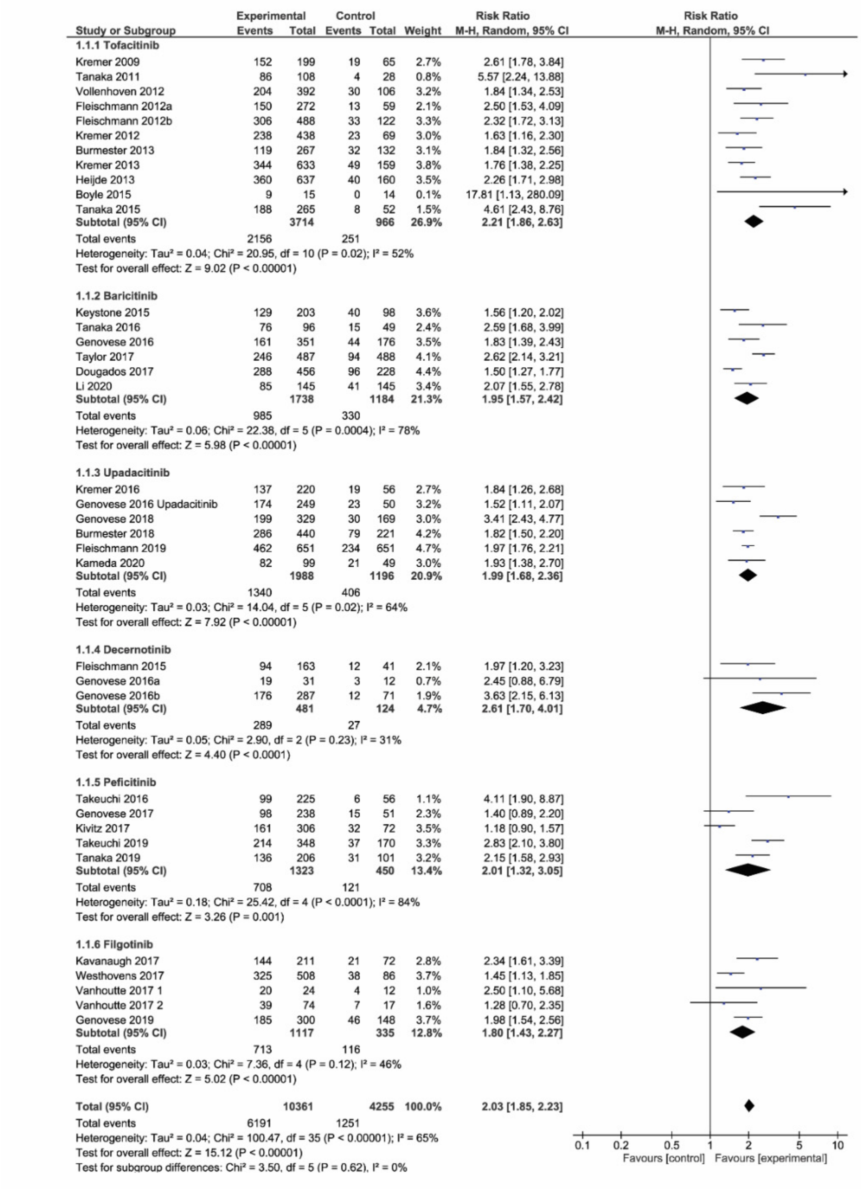

***Figure S2** Forest plot of the effect of JAKinibs on ACR50 (American College of Rheumatology 50 response rates) verse placebo. Randomized-effects model. RR=Relative Risks.*

Study or Subgroup	Experimental Events	Experimental Total	Control Events	Control Total	Weight	Risk Ratio M-H, Random, 95% CI
**1.2.1 Tofacitinib**						
Kremer 2009	84	199	3	65	1.7%	9.15 [2.99, 27.95]
Tanaka 2011	52	108	3	28	1.8%	4.49 [1.52, 13.33]
Vollenhoven 2012	152	392	11	106	3.6%	3.74 [2.11, 6.63]
Fleischmann 2012a	91	272	6	59	2.7%	3.29 [1.51, 7.15]
Fleischmann 2012b	210	488	39	122	5.2%	1.35 [1.02, 1.78]
Kremer 2012	206	438	15	69	4.2%	2.16 [1.37, 3.42]
Burmester 2013	102	267	11	132	3.6%	4.58 [2.55, 8.24]
Kremer 2013	218	633	20	159	4.4%	2.74 [1.79, 4.18]
Heijde 2013	242	637	13	160	3.9%	4.68 [2.75, 7.95]
Boyle 2015	6	15	0	14	0.4%	12.19 [0.75, 198.20]
Tanaka 2015	78	265	3	52	1.7%	5.10 [1.67, 15.55]
**Subtotal (95% CI)**		**3714**		**966**	**33.2%**	**3.43 [2.30, 5.12]**
Total events	1441		124			
Heterogeneity: Tau² = 0.30; Chi² = 44.70, df = 10 (P < 0.00001); I² = 78%						
Test for overall effect: Z = 6.06 (P < 0.00001)						
**1.2.2 Baricitinib**						
Keystone 2015	64	203	8	98	3.1%	3.86 [1.93, 7.73]
Tanaka 2016	57	96	2	49	1.3%	14.55 [3.71, 57.10]
Genovese 2016	92	351	23	176	4.5%	2.01 [1.32, 3.05]
Taylor 2017	246	487	94	488	5.6%	2.62 [2.14, 3.21]
Dougados 2017	195	456	49	228	5.3%	1.99 [1.52, 2.61]
Li 2020	44	145	12	145	3.5%	3.67 [2.02, 6.65]
**Subtotal (95% CI)**		**1738**		**1184**	**23.2%**	**2.73 [2.03, 3.66]**
Total events	698		188			
Heterogeneity: Tau² = 0.07; Chi² = 13.94, df = 5 (P = 0.02); I² = 64%						
Test for overall effect: Z = 6.71 (P < 0.00001)						
**1.2.3 Upadacitinib**						
Kremer 2016	77	220	9	56	0.0%	0.58 [0.44, 0.76]
Genovese 2016 Upadacitinib	106	249	9	50	3.5%	2.37 [1.29, 4.35]
Genovese 2018	99	329	14	169	3.9%	3.63 [2.14, 6.16]
Burmester 2018	178	440	33	221	4.9%	2.71 [1.94, 3.79]
Fleischmann 2019	293	651	98	651	5.6%	2.99 [2.44, 3.66]
Kameda 2020	57	99	8	49	0.0%	3.53 [1.83, 6.80]
**Subtotal (95% CI)**		**1669**		**1091**	**17.8%**	**2.93 [2.50, 3.43]**
Total events	676		154			
Heterogeneity: Tau² = 0.00; Chi² = 1.36, df = 3 (P = 0.71); I² = 0%						
Test for overall effect: Z = 13.31 (P < 0.00001)						
**1.2.4 Decernotinib**						
Fleischmann 2015	55	163	3	41	1.7%	4.61 [1.52, 14.00]
Genovese 2016a	12	31	1	12	0.7%	4.65 [0.68, 31.94]
Genovese 2016b	97	287	5	71	2.4%	4.80 [2.03, 11.35]
**Subtotal (95% CI)**		**481**		**124**	**4.9%**	**4.72 [2.48, 8.96]**
Total events	164		9			
Heterogeneity: Tau² = 0.00; Chi² = 0.00, df = 2 (P = 1.00); I² = 0%						
Test for overall effect: Z = 4.74 (P < 0.00001)						
**1.2.5 Peficitinib**						
Takeuchi 2016	43	225	3	56	1.7%	3.57 [1.15, 11.08]
Genovese 2017	57	238	5	51	2.4%	2.44 [1.03, 5.79]
Kivitz 2017	95	306	19	72	4.5%	1.18 [0.77, 1.79]
Takeuchi 2019	132	348	13	170	3.8%	4.96 [2.89, 8.51]
Tanaka 2019	75	206	9	101	3.3%	4.09 [2.13, 7.82]
**Subtotal (95% CI)**		**1323**		**450**	**15.6%**	**2.84 [1.42, 5.70]**
Total events	402		49			
Heterogeneity: Tau² = 0.49; Chi² = 22.56, df = 4 (P = 0.0002); I² = 82%						
Test for overall effect: Z = 2.94 (P = 0.003)						
**1.2.6 Filgotinib**						
Kavanaugh 2017	79	211	6	72	2.7%	4.49 [2.05, 9.86]
Westhovens 2017	183	508	2	86	1.3%	15.49 [3.92, 61.24]
Vanhoutte 2017 1	6	24	1	12	0.7%	3.00 [0.41, 22.18]
Vanhoutte 2017 2	17	74	1	17	0.7%	3.91 [0.56, 27.36]
**Subtotal (95% CI)**		**817**		**187**	**5.3%**	**5.56 [2.79, 11.06]**
Total events	285		10			
Heterogeneity: Tau² = 0.06; Chi² = 3.37, df = 3 (P = 0.34); I² = 11%						
Test for overall effect: Z = 4.89 (P < 0.00001)						
**Total (95% CI)**		**9742**		**4002**	**100.0%**	**3.14 [2.64, 3.73]**
Total events	3666		534			
Heterogeneity: Tau² = 0.13; Chi² = 97.17, df = 32 (P < 0.00001); I² = 67%						
Test for overall effect: Z = 12.98 (P < 0.00001)						
Test for subgroup differences: Chi² = 5.89, df = 5 (P = 0.32), I² = 15.2%						

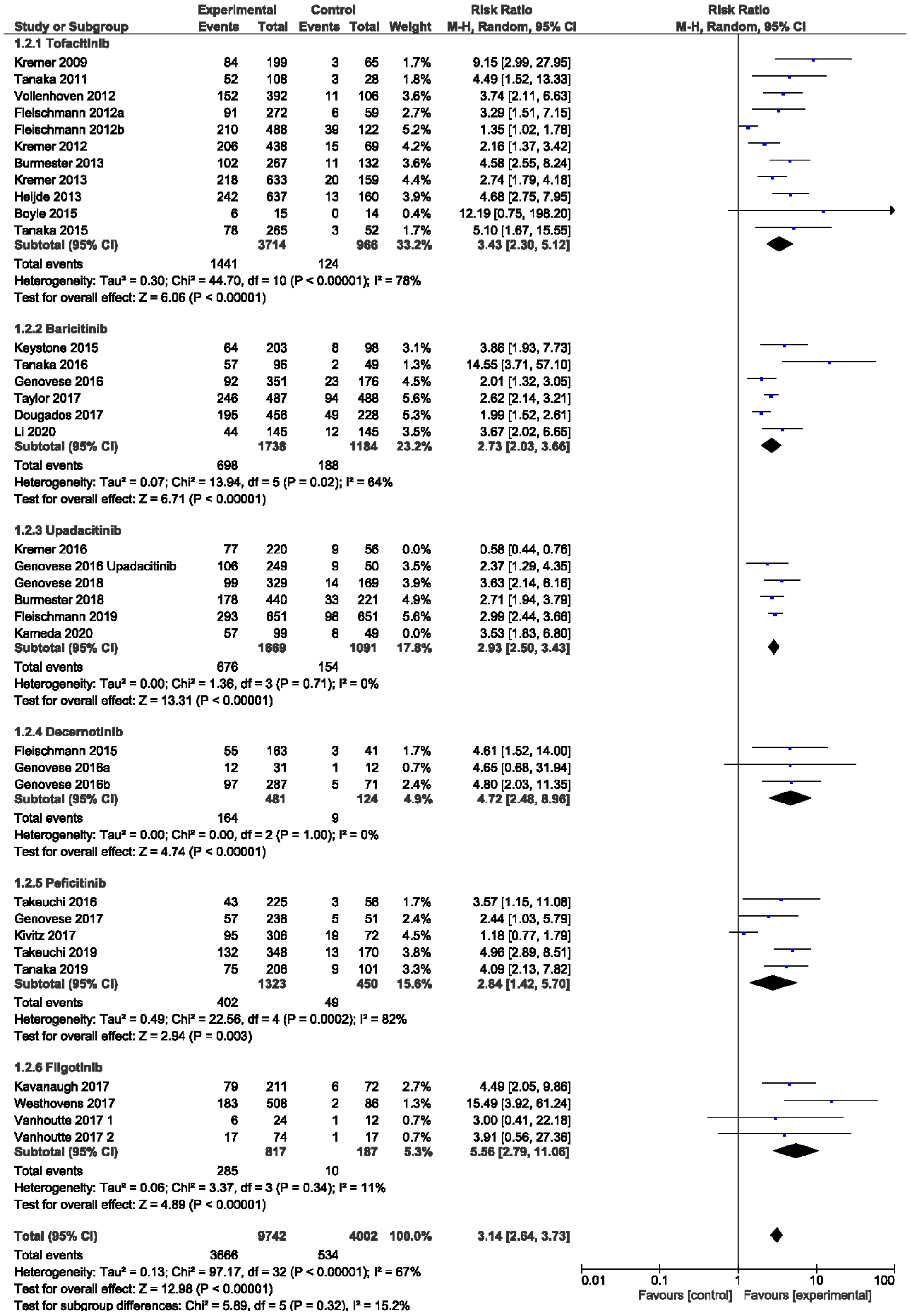

Figure S3 *Forest plot of the effect of JAKinibs on ACR70 (American College of Rheumatology 70 response rates) verse placebo. Randomized-effects model. RR=Relative Risks.*

Study or Subgroup	Experimental Events	Experimental Total	Control Events	Control Total	Weight	Risk Ratio M-H, Random, 95% CI
**1.3.1 Tofacitinib**						
Boyle 2015	1	15	0	14	0.6%	2.81 [0.12, 63.83]
Burmester 2013	32	267	2	132	2.2%	7.91 [1.92, 32.51]
Fleischmann 2012a	45	272	8	59	4.7%	1.22 [0.61, 2.45]
Fleischmann 2012b	122	488	22	122	6.2%	1.39 [0.92, 2.09]
Heijde 2013	117	637	2	160	2.3%	14.69 [3.67, 58.81]
Kremer 2009	41	199	2	65	2.2%	6.70 [1.67, 26.92]
Kremer 2012	94	438	5	69	3.9%	2.96 [1.25, 7.02]
Kremer 2013	91	633	5	159	3.8%	4.57 [1.89, 11.06]
Tanaka 2011	21	108	1	28	1.3%	5.44 [0.76, 38.75]
Tanaka 2015	68	265	1	52	1.3%	13.34 [1.89, 93.96]
Vollenhoven 2012	79	392	3	106	2.9%	7.12 [2.29, 22.10]
**Subtotal (95% CI)**		**3714**		**966**	**31.6%**	**4.15 [2.21, 7.80]**
Total events	711		51			
Heterogeneity: Tau² = 0.70; Chi² = 37.85, df = 10 (P < 0.0001); I² = 74%						
Test for overall effect: Z = 4.43 (P < 0.00001)						
**1.3.2 Baricitinib**						
Dougados 2017	113	456	18	228	5.9%	3.14 [1.96, 5.03]
Genovese 2016	53	351	6	176	4.1%	4.43 [1.94, 10.10]
Keystone 2015	29	203	2	98	2.2%	7.00 [1.70, 28.74]
Li 2020	14	145	1	145	1.3%	14.00 [1.87, 105.08]
Tanaka 2016	22	96	0	49	0.7%	23.20 [1.44, 374.49]
Taylor 2017	145	487	39	488	6.6%	3.73 [2.68, 5.18]
**Subtotal (95% CI)**		**1738**		**1184**	**20.8%**	**3.81 [2.97, 4.89]**
Total events	376		66			
Heterogeneity: Tau² = 0.00; Chi² = 4.93, df = 5 (P = 0.42); I² = 0%						
Test for overall effect: Z = 10.49 (P < 0.00001)						
**1.3.3 Upadacitinib**						
Burmester 2018	46	220	2	56	2.3%	5.85 [1.47, 23.39]
Fleischmann 2019	57	249	3	50	3.0%	3.82 [1.24, 11.70]
Genovese 2016 Upadacitinib	43	329	7	169	4.3%	3.16 [1.45, 6.86]
Genovese 2018	106	440	13	221	5.4%	4.10 [2.36, 7.12]
Kameda 2020	189	651	39	651	6.6%	4.85 [3.49, 6.72]
Kremer 2016	31	99	1	49	1.3%	15.34 [2.16, 109.11]
**Subtotal (95% CI)**		**1988**		**1196**	**22.9%**	**4.53 [3.53, 5.83]**
Total events	472		65			
Heterogeneity: Tau² = 0.00; Chi² = 2.86, df = 5 (P = 0.72); I² = 0%						
Test for overall effect: Z = 11.80 (P < 0.00001)						
**1.3.4 Decernotinib**						
Fleischmann 2015	24	163	1	41	1.3%	6.04 [0.84, 43.32]
Genovese 2016a	5	31	1	12	1.2%	1.94 [0.25, 14.90]
Genovese 2016b	38	287	2	71	2.2%	4.70 [1.16, 19.02]
**Subtotal (95% CI)**		**481**		**124**	**4.8%**	**4.06 [1.50, 10.98]**
Total events	67		4			
Heterogeneity: Tau² = 0.00; Chi² = 0.73, df = 2 (P = 0.69); I² = 0%						
Test for overall effect: Z = 2.76 (P = 0.006)						
**1.3.5 Peficitinib**						
Genovese 2017	17	225	1	56	1.3%	4.23 [0.58, 31.12]
Kivitz 2017	31	238	4	51	3.4%	1.66 [0.61, 4.50]
Takeuchi 2016	47	306	8	72	4.7%	1.38 [0.68, 2.80]
Takeuchi 2019	62	348	4	170	3.4%	7.57 [2.80, 20.47]
Tanaka 2019	42	206	1	101	1.3%	20.59 [2.88, 147.49]
**Subtotal (95% CI)**		**1323**		**450**	**14.1%**	**3.64 [1.32, 10.05]**
Total events	199		18			
Heterogeneity: Tau² = 0.90; Chi² = 15.02, df = 4 (P = 0.005); I² = 73%						
Test for overall effect: Z = 2.49 (P = 0.01)						
**1.3.6 Filgotinib**						
Kavanaugh 2017	27	211	1	72	1.3%	9.21 [1.27, 66.59]
Westhovens 2017	94	508	7	86	4.5%	2.27 [1.09, 4.73]
**Subtotal (95% CI)**		**719**		**158**	**5.8%**	**3.41 [0.94, 12.40]**
Total events	121		8			
Heterogeneity: Tau² = 0.47; Chi² = 1.81, df = 1 (P = 0.18); I² = 45%						
Test for overall effect: Z = 1.87 (P = 0.06)						
**Total (95% CI)**		**9963**		**4078**	**100.0%**	**3.87 [3.02, 4.97]**
Total events	1946		212			
Heterogeneity: Tau² = 0.22; Chi² = 72.37, df = 32 (P < 0.0001); I² = 56%						
Test for overall effect: Z = 10.63 (P < 0.00001)						
Test for subgroup differences: Chi² = 1.07, df = 5 (P = 0.96), I² = 0%						

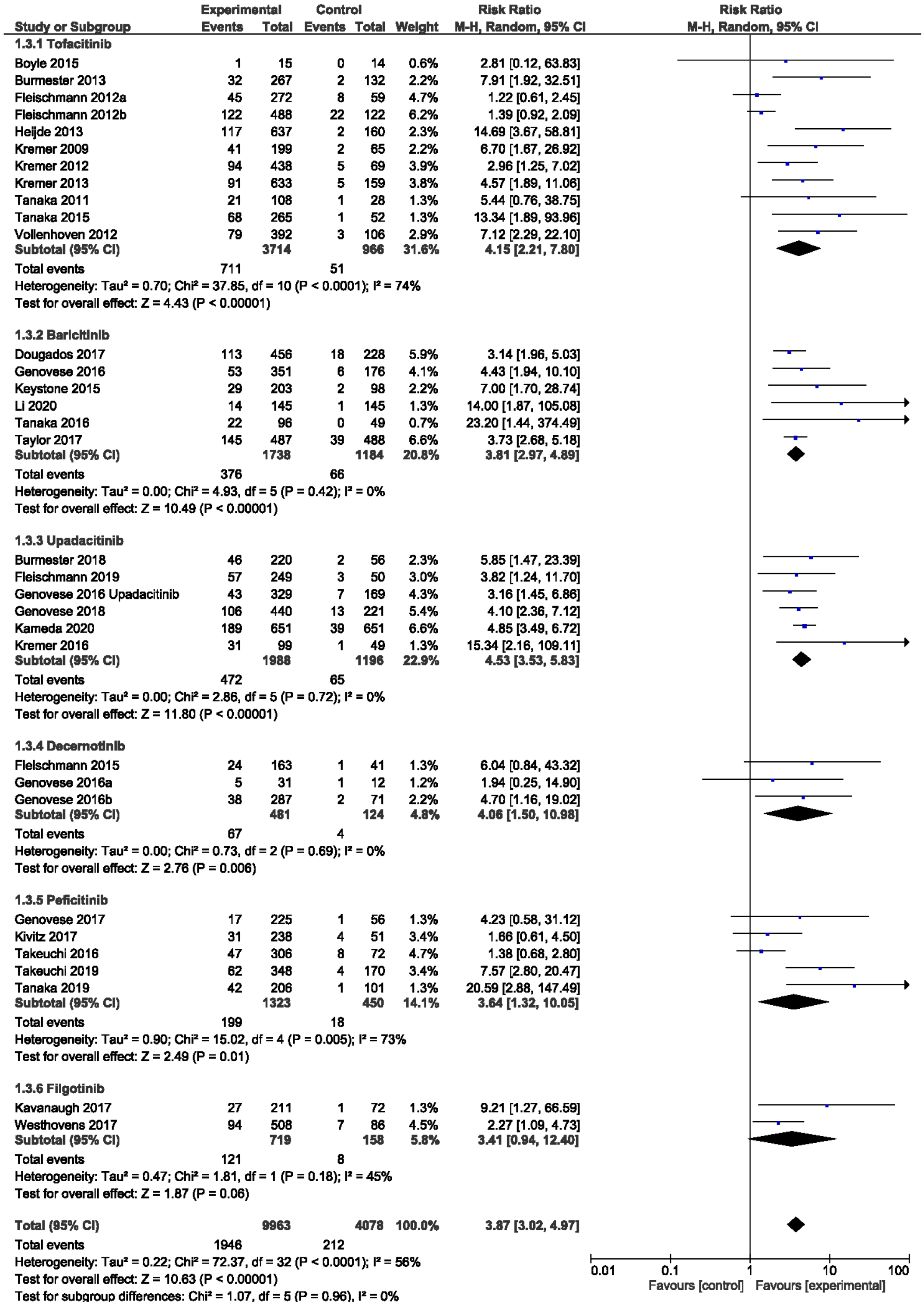

Figure S4 *Forest plot of the effect of JAKinibs on HAQ-ID (Health Assessment Questionnaire–Disability Index) verse placebo. Randomized-effects model. MD= mean difference.*

Study or Subgroup	Experimental Mean	Experimental SD	Experimental Total	Control Mean	Control SD	Control Total	Weight	Mean Difference IV, Random, 95% CI
**2.1.1 Tofacitinib**								
Kremer 2009	-0.67	0.6	169	-0.3	0.5	65	3.6%	-0.37 [-0.52, -0.22]
Tanaka 2011	-0.46	2.51	108	-0.05	2.52	28	0.1%	-0.41 [-1.46, 0.64]
Fleischmann 2012a	-0.54	0.59	272	-0.25	0.61	59	2.9%	-0.29 [-0.46, -0.12]
Kremer 2012	-0.45	0.54	438	-0.16	0.5	69	5.1%	-0.29 [-0.42, -0.16]
Heijde 2013	-0.55	0.54	637	-0.17	0.63	160	7.4%	-0.38 [-0.49, -0.27]
Kremer 2013	-0.48	0.69	633	-0.16	0.65	160	6.4%	-0.32 [-0.43, -0.21]
Vollenhoven 2012	-0.58	0.63	392	-0.24	0.68	108	4.1%	-0.34 [-0.48, -0.20]
**Subtotal (95% CI)**			**2649**			**649**	**29.7%**	**-0.34 [-0.39, -0.28]**
Heterogeneity: Tau² = 0.00; Chi² = 1.72, df = 6 (P = 0.94); I² = 0%								
Test for overall effect: Z = 12.39 (P < 0.00001)								
**2.1.2 Baricitinib**								
Keystone 2015	-0.31	0.67	203	-0.1	0.7	98	3.0%	-0.21 [-0.38, -0.04]
Genovese 2016	-0.4	0.57	351	-0.15	0.57	176	7.9%	-0.25 [-0.35, -0.15]
**Subtotal (95% CI)**			**554**			**274**	**10.9%**	**-0.24 [-0.33, -0.15]**
Heterogeneity: Tau² = 0.00; Chi² = 0.16, df = 1 (P = 0.69); I² = 0%								
Test for overall effect: Z = 5.34 (P < 0.00001)								
**2.1.3 Upadacitinib**								
Genovese 2016 Upadacitinib	-0.66	0.66	249	-0.4	0.7	50	1.9%	-0.26 [-0.47, -0.05]
Genovese 2018	-0.43	0.63	329	-0.16	0.6	169	6.5%	-0.27 [-0.38, -0.16]
Burmester 2018	-0.58	0.6	440	-0.26	0.6	221	8.9%	-0.32 [-0.42, -0.22]
Fleischmann 2019	-0.6	0.6	651	-0.28	0.6	651	19.7%	-0.32 [-0.39, -0.25]
Kameda 2020	-0.47	0.65	99	-0.1	0.7	49	1.5%	-0.37 [-0.60, -0.14]
**Subtotal (95% CI)**			**1768**			**1140**	**38.6%**	**-0.31 [-0.36, -0.26]**
Heterogeneity: Tau² = 0.00; Chi² = 1.08, df = 4 (P = 0.90); I² = 0%								
Test for overall effect: Z = 13.06 (P < 0.00001)								
**2.1.4 Decernotinib**								
Fleischmann 2015	-0.48	0.52	163	-0.12	0.51	41	2.7%	-0.36 [-0.54, -0.18]
Genovese 2016b	-0.71	0.59	287	-0.6	0.76	71	2.3%	-0.11 [-0.30, 0.08]
**Subtotal (95% CI)**			**450**			**112**	**5.1%**	**-0.24 [-0.48, 0.01]**
Heterogeneity: Tau² = 0.02; Chi² = 3.60, df = 1 (P = 0.06); I² = 72%								
Test for overall effect: Z = 1.90 (P = 0.06)								
**2.1.5 Peficitinib**								
Takeuchi 2016	-0.06	0.67	225	0.16	0.68	56	2.1%	-0.22 [-0.42, -0.02]
**Subtotal (95% CI)**			**225**			**56**	**2.1%**	**-0.22 [-0.42, -0.02]**
Heterogeneity: Not applicable								
Test for overall effect: Z = 2.17 (P = 0.03)								
**2.1.6 Filgotinib**								
Kavanaugh 2017	-0.69	0.67	211	-0.226	0.6	72	3.1%	-0.46 [-0.63, -0.30]
Westhovens 2017	-0.67	0.65	508	-0.383	0.64	86	3.9%	-0.29 [-0.43, -0.14]
Genovese 2019	-0.51	0.6	300	-0.23	0.55	148	6.7%	-0.28 [-0.39, -0.17]
**Subtotal (95% CI)**			**1019**			**306**	**13.7%**	**-0.33 [-0.44, -0.22]**
Heterogeneity: Tau² = 0.00; Chi² = 3.59, df = 2 (P = 0.17); I² = 44%								
Test for overall effect: Z = 6.04 (P < 0.00001)								
**Total (95% CI)**			**6665**			**2537**	**100.0%**	**-0.31 [-0.34, -0.28]**
Heterogeneity: Tau² = 0.00; Chi² = 15.45, df = 19 (P = 0.69); I² = 0%								
Test for overall effect: Z = 20.78 (P < 0.00001)								
Test for subgroup differences: Chi² = 4.73, df = 5 (P = 0.45), I² = 0%								

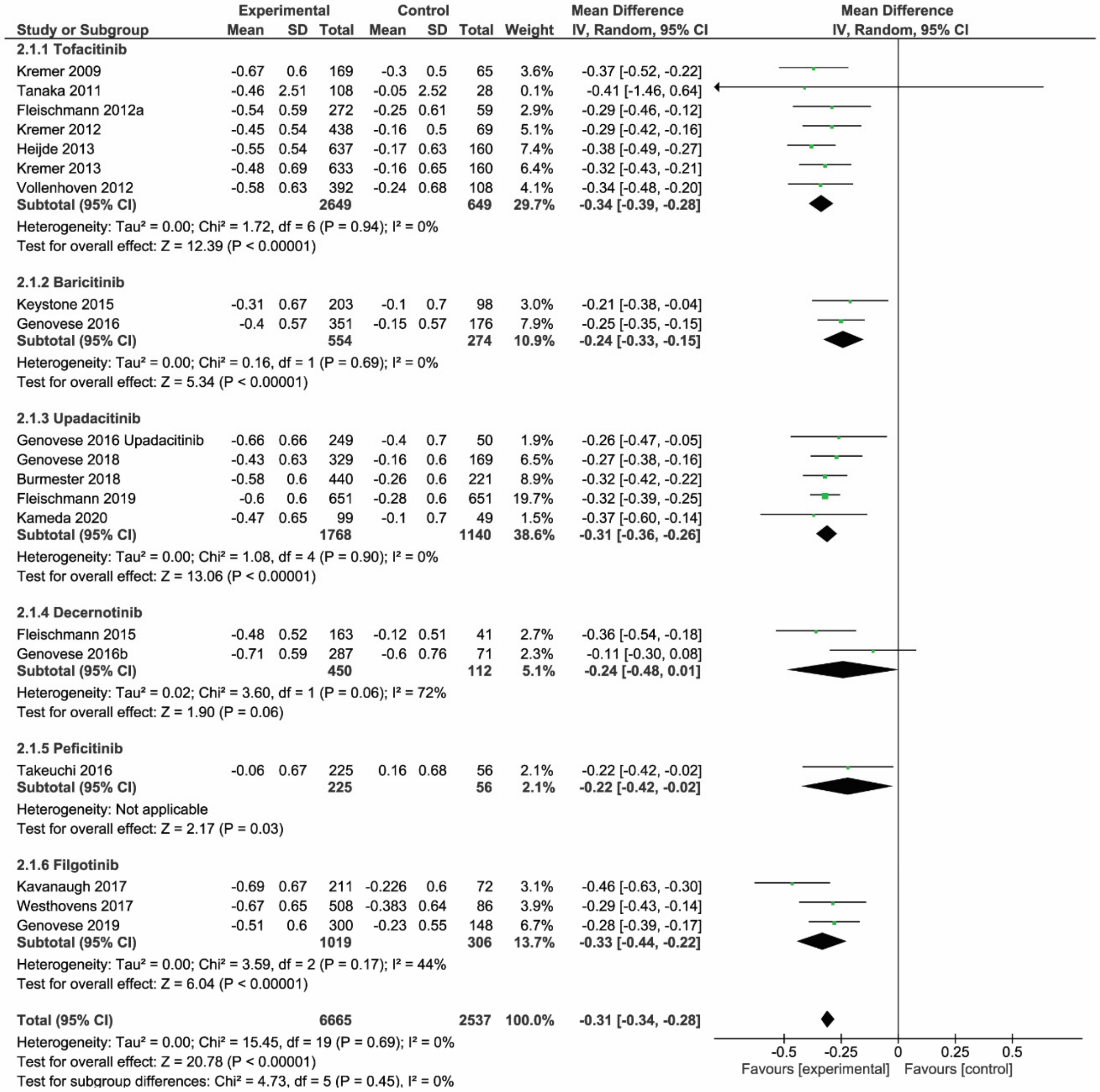

Figure S5 *Forest plot of the effect of JAKinibs on AEs. Randomized-effects model. RR=Relative Risks.*

Study or Subgroup	Experimental Events	Experimental Total	Control Events	Control Total	Weight	Risk Ratio M-H, Random, 95% CI
**3.1.1 Tofacitinib**						
Kremer 2009	141	199	19	38	1.4%	1.42 [1.02, 1.97]
Vollenhoven 2012	210	405	51	108	2.9%	1.10 [0.88, 1.37]
Fleischmann 2012a	146	272	29	59	1.9%	1.09 [0.82, 1.45]
Fleischmann 2012b	263	488	67	122	3.9%	0.98 [0.82, 1.18]
Kremer 2012	292	438	41	69	3.2%	1.12 [0.91, 1.38]
Heijde 2013	554	637	120	160	8.1%	1.16 [1.06, 1.27]
Kremer 2013	83	633	26	159	1.0%	0.80 [0.53, 1.20]
Burmester 2013	147	267	75	132	3.8%	0.97 [0.81, 1.16]
Boyle 2015	7	15	10	14	0.4%	0.65 [0.35, 1.23]
Tanaka 2015	133	265	23	52	1.5%	1.13 [0.82, 1.58]
Kremer 2015	42	97	26	51	1.3%	0.85 [0.60, 1.21]
**Subtotal (95% CI)**		**3716**		**964**	**29.2%**	**1.06 [0.98, 1.15]**
Total events	2018		487			
Heterogeneity: Tau² = 0.01; Chi² = 14.10, df = 10 (P = 0.17); I² = 29%						
Test for overall effect: Z = 1.41 (P = 0.16)						
**3.1.2 Baricitinib**						
Keystone 2015	92	203	40	98	1.9%	1.11 [0.84, 1.47]
Tanaka 2016	54	96	26	49	1.5%	1.06 [0.77, 1.46]
Genovese 2016	260	351	112	176	6.0%	1.16 [1.02, 1.32]
Dougados 2017	316	456	161	228	7.5%	0.98 [0.88, 1.09]
Taylor 2017	347	487	295	488	8.3%	1.18 [1.08, 1.29]
**Subtotal (95% CI)**		**1593**		**1039**	**25.2%**	**1.10 [1.01, 1.21]**
Total events	1069		634			
Heterogeneity: Tau² = 0.00; Chi² = 7.68, df = 4 (P = 0.10); I² = 48%						
Test for overall effect: Z = 2.07 (P = 0.04)						
**3.1.3 Upadacitinib**						
Kremer 2016	133	220	25	56	1.6%	1.35 [0.99, 1.85]
Genovese 2016	114	249	13	50	0.7%	1.76 [1.08, 2.87]
Burmester 2018	243	440	108	221	4.6%	1.13 [0.96, 1.32]
Genovese 2018	202	329	95	169	4.6%	1.09 [0.93, 1.28]
Fleischmann 2019	417	650	347	652	8.2%	1.21 [1.10, 1.32]
Kameda 2020	65	99	24	49	1.5%	1.34 [0.97, 1.84]
**Subtotal (95% CI)**		**1987**		**1197**	**21.3%**	**1.19 [1.11, 1.28]**
Total events	1174		612			
Heterogeneity: Tau² = 0.00; Chi² = 5.36, df = 5 (P = 0.37); I² = 7%						
Test for overall effect: Z = 4.71 (P < 0.00001)						
**3.1.4 Decernotinib**						
Fleischmann 2015	77	163	19	41	1.2%	1.02 [0.71, 1.47]
Genovese 2016a	25	31	5	12	0.4%	1.94 [0.97, 3.86]
Genovese 2016b	172	287	30	71	1.8%	1.42 [1.06, 1.89]
**Subtotal (95% CI)**		**481**		**124**	**3.4%**	**1.32 [0.97, 1.78]**
Total events	274		54			
Heterogeneity: Tau² = 0.03; Chi² = 3.32, df = 2 (P = 0.19); I² = 40%						
Test for overall effect: Z = 1.79 (P = 0.07)						
**3.1.5 Peficitinib**						
Takeuchi 2016	144	225	36	56	2.9%	1.00 [0.80, 1.24]
Genovese 2017	99	238	22	51	1.3%	0.96 [0.68, 1.37]
Kivitz 2017	146	306	34	72	2.0%	1.01 [0.77, 1.32]
Takeuchi 2019	193	348	84	170	3.9%	1.12 [0.94, 1.34]
Tanaka 2019	114	206	54	101	2.9%	1.04 [0.83, 1.29]
**Subtotal (95% CI)**		**1323**		**450**	**13.0%**	**1.04 [0.94, 1.16]**
Total events	696		230			
Heterogeneity: Tau² = 0.00; Chi² = 1.07, df = 4 (P = 0.90); I² = 0%						
Test for overall effect: Z = 0.82 (P = 0.41)						
**3.1.6 Filgotinib**						
Kavanaugh 2017	82	211	28	72	1.4%	1.00 [0.71, 1.40]
Westhovens 2017	248	472	32	56	2.4%	0.92 [0.72, 1.17]
Vanhoutte 2017 1	5	24	4	12	0.1%	0.63 [0.20, 1.91]
Vanhoutte 2017 2	10	74	1	17	0.0%	2.30 [0.32, 16.75]
Genovese 2019	159	300	80	148	3.8%	0.98 [0.82, 1.18]
**Subtotal (95% CI)**		**1081**		**305**	**7.8%**	**0.96 [0.84, 1.10]**
Total events	504		145			
Heterogeneity: Tau² = 0.00; Chi² = 1.54, df = 4 (P = 0.82); I² = 0%						
Test for overall effect: Z = 0.57 (P = 0.57)						
**Total (95% CI)**		**10181**		**4079**	**100.0%**	**1.10 [1.05, 1.14]**
Total events	5735		2162			
Heterogeneity: Tau² = 0.00; Chi² = 45.36, df = 34 (P = 0.09); I² = 25%						
Test for overall effect: Z = 4.33 (P < 0.0001)						
Test for subgroup differences: Chi² = 11.56, df = 5 (P = 0.04), I² = 56.8%						

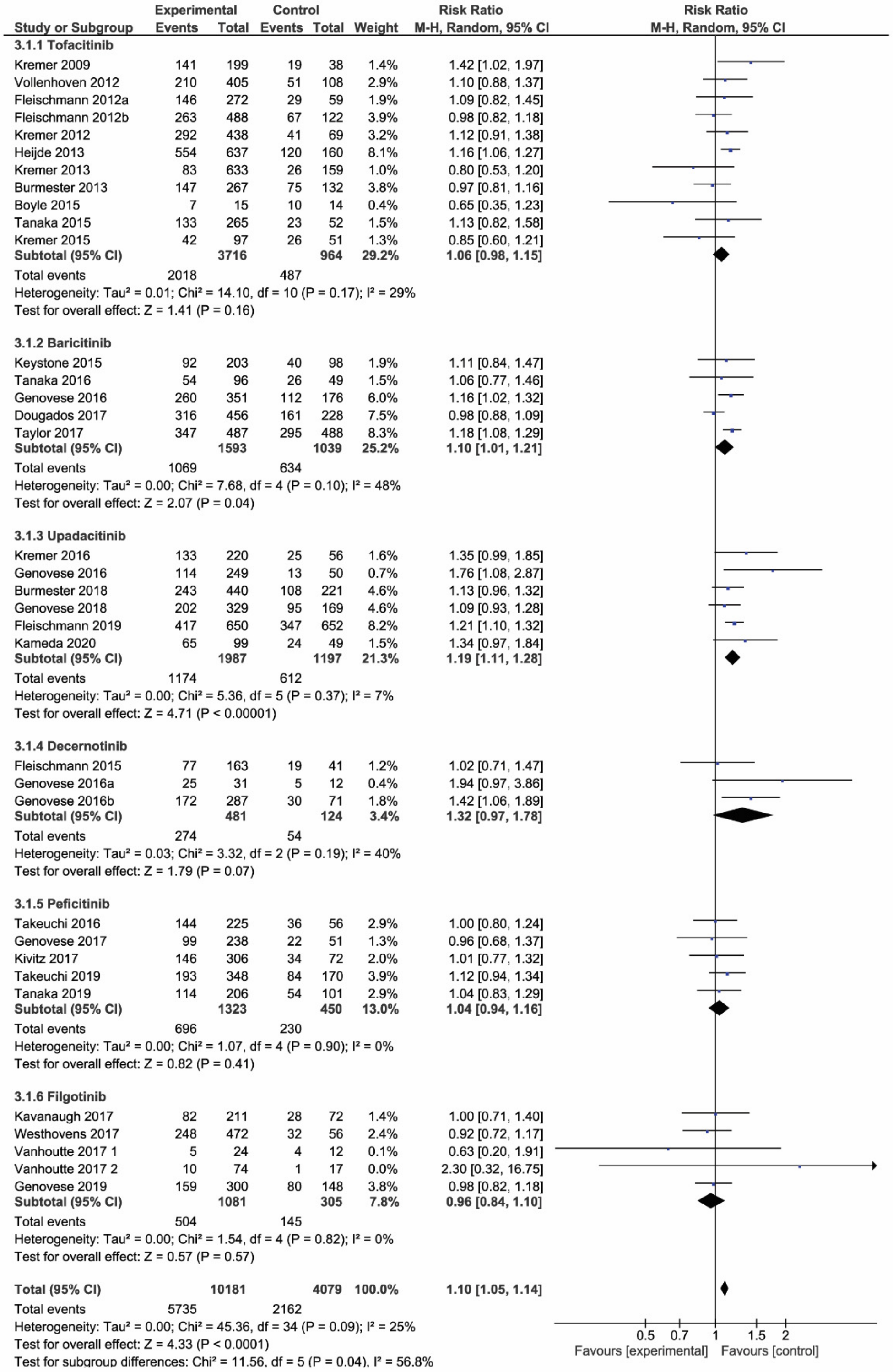

Figure S6 *Forest plot of the effect of JAKinibs on SAEs. Randomized-effects model. RR=Relative Risks.*

Study or Subgroup	Experimental Events	Experimental Total	Control Events	Control Total	Weight	Risk Ratio M-H, Random, 95% CI
**4.1.1 Tofacitinib**						
Kremer 2009	2	199	1	65	0.7%	0.65 [0.06, 7.09]
Tanaka 2011	5	108	0	28	0.5%	2.93 [0.17, 51.41]
Vollenhoven 2012	22	405	2	108	2.0%	2.93 [0.70, 12.28]
Heijde 2013	22	637	4	160	3.7%	1.38 [0.48, 3.95]
Kremer 2013	2	633	0	159	0.4%	1.26 [0.06, 26.15]
Burmester 2013	5	267	6	132	3.0%	0.41 [0.13, 1.33]
Boyle 2015	7	15	10	14	10.2%	0.65 [0.35, 1.23]
Tanaka 2015	8	265	1	52	1.0%	1.57 [0.20, 12.29]
Kremer 2015	3	97	4	51	1.9%	0.39 [0.09, 1.69]
Fleischmann 2012a	8	272	2	59	1.8%	0.87 [0.19, 3.98]
Fleischmann 2012b	6	488	6	122	3.3%	0.25 [0.08, 0.76]
**Subtotal (95% CI)**		**3386**		**950**	**28.6%**	**0.74 [0.47, 1.18]**
Total events	90		36			
Heterogeneity: Tau² = 0.12; Chi² = 12.54, df = 10 (P = 0.25); I² = 20%						
Test for overall effect: Z = 1.27 (P = 0.20)						
**4.1.2 Baricitinib**						
Keystone 2015	4	203	3	98	1.9%	0.64 [0.15, 2.82]
Tanaka 2016	2	96	1	49	0.7%	1.02 [0.09, 10.98]
Genovese 2016	25	351	13	176	9.9%	0.96 [0.51, 1.84]
Taylor 2017	23	487	22	488	12.6%	1.05 [0.59, 1.85]
Dougados 2017	18	456	11	228	7.6%	0.82 [0.39, 1.70]
Li 2020	1	145	2	145	0.7%	0.50 [0.05, 5.45]
**Subtotal (95% CI)**		**1738**		**1184**	**33.4%**	**0.92 [0.65, 1.31]**
Total events	73		52			
Heterogeneity: Tau² = 0.00; Chi² = 0.80, df = 5 (P = 0.98); I² = 0%						
Test for overall effect: Z = 0.44 (P = 0.66)						
**4.1.3 Upadacitinib**						
Kremer 2016	5	220	1	56	0.9%	1.27 [0.15, 10.68]
Genovese 2016 Upadacitinib	8	249	0	50	0.5%	3.47 [0.20, 59.13]
Genovese 2018	20	329	0	169	0.5%	21.12 [1.29, 347.09]
Burmester 2018	15	440	5	221	4.1%	1.51 [0.55, 4.09]
Fleischmann 2019	24	650	19	652	11.7%	1.27 [0.70, 2.29]
Kameda 2020	6	99	0	49	0.5%	6.50 [0.37, 113.09]
**Subtotal (95% CI)**		**1987**		**1197**	**18.3%**	**1.72 [0.92, 3.25]**
Total events	78		25			
Heterogeneity: Tau² = 0.12; Chi² = 6.07, df = 5 (P = 0.30); I² = 18%						
Test for overall effect: Z = 1.69 (P = 0.09)						
**4.1.4 Decernotinib**						
Fleischmann 2015	5	163	0	41	0.5%	2.82 [0.16, 49.94]
Genovese 2016a	2	31	0	12	0.5%	2.03 [0.10, 39.48]
Genovese 2016b	21	287	4	71	3.8%	1.30 [0.46, 3.66]
**Subtotal (95% CI)**		**481**		**124**	**4.8%**	**1.47 [0.58, 3.71]**
Total events	28		4			
Heterogeneity: Tau² = 0.00; Chi² = 0.30, df = 2 (P = 0.86); I² = 0%						
Test for overall effect: Z = 0.82 (P = 0.41)						
**4.1.5 Peficitinib**						
Takeuchi 2016	6	225	1	56	0.9%	1.49 [0.18, 12.15]
Genovese 2017	6	238	1	51	0.9%	1.29 [0.16, 10.45]
Kivitz 2017	3	306	0	72	0.5%	1.66 [0.09, 31.87]
Takeuchi 2019	8	348	4	170	2.9%	0.98 [0.30, 3.20]
Tanaka 2019	5	206	4	101	2.5%	0.61 [0.17, 2.23]
**Subtotal (95% CI)**		**1323**		**450**	**7.7%**	**0.95 [0.46, 1.96]**
Total events	28		10			
Heterogeneity: Tau² = 0.00; Chi² = 0.85, df = 4 (P = 0.93); I² = 0%						
Test for overall effect: Z = 0.15 (P = 0.88)						
**4.1.6 Filgotinib**						
Kavanaugh 2017	4	211	1	72	0.9%	1.36 [0.16, 12.01]
Westhovens 2017	10	472	4	56	3.2%	0.30 [0.10, 0.91]
Genovese 2019	10	300	4	148	3.1%	1.23 [0.39, 3.87]
**Subtotal (95% CI)**		**983**		**276**	**7.2%**	**0.70 [0.24, 2.07]**
Total events	24		9			
Heterogeneity: Tau² = 0.41; Chi² = 3.67, df = 2 (P = 0.16); I² = 46%						
Test for overall effect: Z = 0.64 (P = 0.52)						
**Total (95% CI)**		**9898**		**4181**	**100.0%**	**0.94 [0.77, 1.15]**
Total events	321		136			
Heterogeneity: Tau² = 0.00; Chi² = 32.18, df = 33 (P = 0.51); I² = 0%						
Test for overall effect: Z = 0.57 (P = 0.57)						
Test for subgroup differences: Chi² = 5.68, df = 5 (P = 0.34), I² = 11.9%						

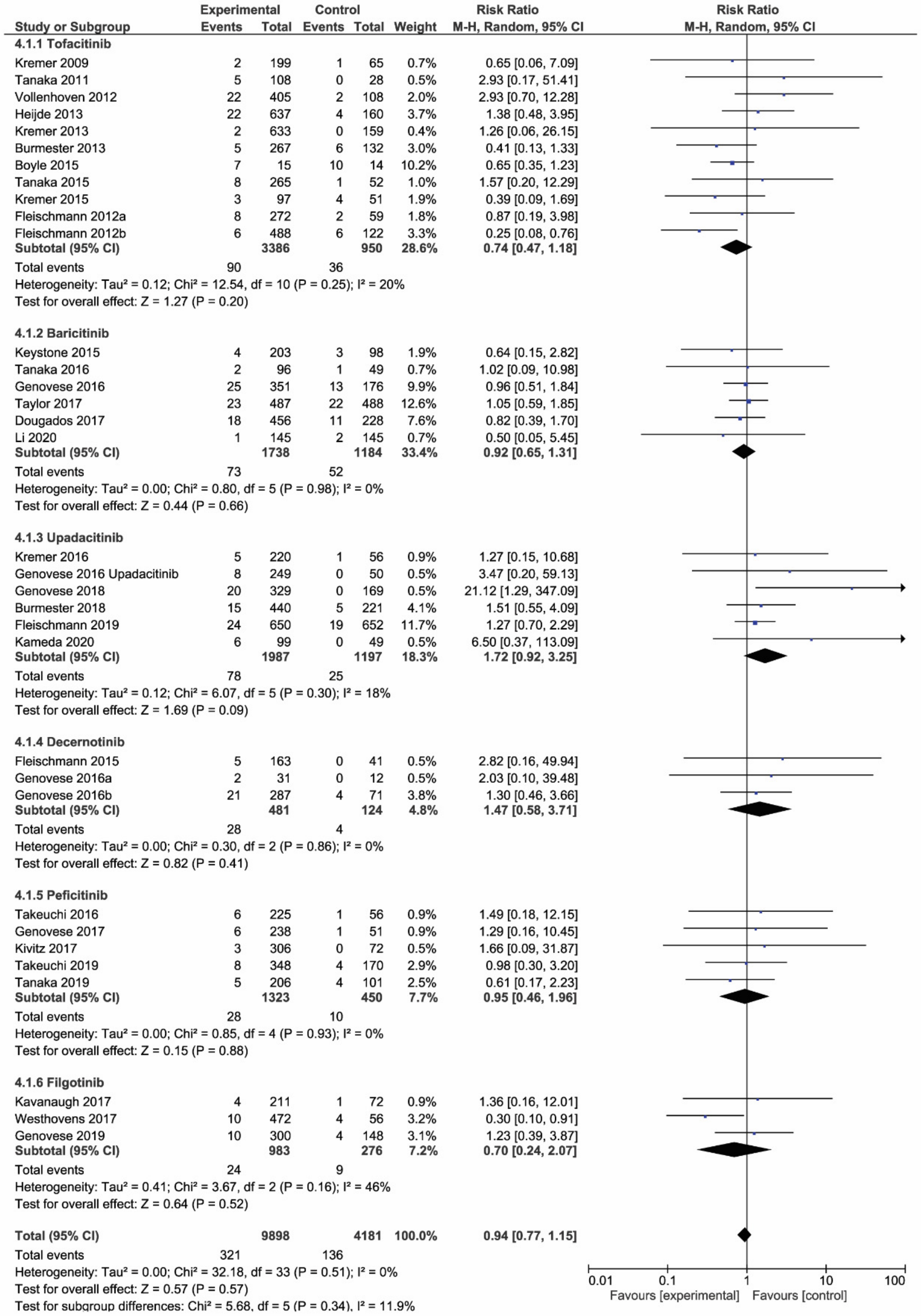

***Figure S7** Forest plot of the effect of JAKinibs on infections. Randomized-effects model. RR=Relative Risks.*

Study or Subgroup	Experimental Events	Experimental Total	Control Events	Control Total	Weight	Risk Ratio M-H, Random, 95% CI
**5.1.1 Tofacitinib**						
Kremer 2009	29	199	9	65	1.2%	1.05 [0.53, 2.11]
Fleischmann 2012a	80	272	12	59	2.1%	1.45 [0.84, 2.48]
Kremer 2012	96	438	7	69	1.1%	2.16 [1.05, 4.46]
Tanaka 2011	25	108	6	28	1.0%	1.08 [0.49, 2.38]
**Subtotal (95% CI)**		**1017**		**221**	**5.4%**	**1.39 [1.00, 1.94]**
Total events	230		34			
Heterogeneity: Tau² = 0.00; Chi² = 2.51, df = 3 (P = 0.47); I² = 0%						
Test for overall effect: Z = 1.94 (P = 0.05)						
**5.1.2 Baricitinib**						
Tanaka 2016	22	96	11	49	1.5%	1.02 [0.54, 1.93]
Genovese 2016	146	351	55	176	9.4%	1.33 [1.03, 1.71]
Taylor 2017	176	487	134	488	17.2%	1.32 [1.09, 1.59]
Dougados 2017	166	456	79	228	12.9%	1.05 [0.85, 1.30]
Li 2020	41	145	32	145	3.7%	1.28 [0.86, 1.91]
**Subtotal (95% CI)**		**1535**		**1086**	**44.7%**	**1.22 [1.09, 1.37]**
Total events	551		311			
Heterogeneity: Tau² = 0.00; Chi² = 3.30, df = 4 (P = 0.51); I² = 0%						
Test for overall effect: Z = 3.42 (P = 0.0006)						
**5.1.3 Upadacitinib**						
Kremer 2016	66	220	13	56	2.2%	1.29 [0.77, 2.17]
Genovese 2016 Upadacitinib	49	249	7	50	1.1%	1.41 [0.68, 2.92]
Burmester 2018	133	440	47	221	7.1%	1.42 [1.06, 1.90]
Genovese 2018	109	329	51	169	7.9%	1.10 [0.83, 1.45]
Fleischmann 2019	226	650	154	652	19.9%	1.47 [1.24, 1.75]
Kameda 2020	38	99	11	49	1.8%	1.71 [0.96, 3.05]
**Subtotal (95% CI)**		**1987**		**1197**	**40.0%**	**1.38 [1.22, 1.56]**
Total events	621		283			
Heterogeneity: Tau² = 0.00; Chi² = 3.81, df = 5 (P = 0.58); I² = 0%						
Test for overall effect: Z = 5.14 (P < 0.00001)						
**5.1.4 Decernotinib**						
Fleischmann 2015	28	163	7	41	1.1%	1.01 [0.47, 2.14]
Genovese 2016b	82	287	11	71	1.8%	1.84 [1.04, 3.27]
**Subtotal (95% CI)**		**450**		**112**	**2.9%**	**1.43 [0.80, 2.58]**
Total events	110		18			
Heterogeneity: Tau² = 0.07; Chi² = 1.59, df = 1 (P = 0.21); I² = 37%						
Test for overall effect: Z = 1.20 (P = 0.23)						
**5.1.5 Peficitinib**						
Takeuchi 2016	56	225	12	56	2.0%	1.16 [0.67, 2.01]
Kivitz 2017	31	306	9	72	1.2%	0.81 [0.40, 1.63]
**Subtotal (95% CI)**		**531**		**128**	**3.2%**	**1.01 [0.66, 1.56]**
Total events	87		21			
Heterogeneity: Tau² = 0.00; Chi² = 0.63, df = 1 (P = 0.43); I² = 0%						
Test for overall effect: Z = 0.05 (P = 0.96)						
**5.1.6 Filgotinib**						
Westhovens 2017	34	472	1	56	0.2%	4.03 [0.56, 28.90]
Genovese 2019	63	300	27	148	3.6%	1.15 [0.77, 1.73]
**Subtotal (95% CI)**		**772**		**204**	**3.8%**	**1.50 [0.53, 4.20]**
Total events	97		28			
Heterogeneity: Tau² = 0.31; Chi² = 1.58, df = 1 (P = 0.21); I² = 37%						
Test for overall effect: Z = 0.77 (P = 0.44)						
**Total (95% CI)**		**6292**		**2948**	**100.0%**	**1.29 [1.19, 1.39]**
Total events	1696		695			
Heterogeneity: Tau² = 0.00; Chi² = 17.12, df = 20 (P = 0.65); I² = 0%						
Test for overall effect: Z = 6.46 (P < 0.00001)						
Test for subgroup differences: Chi² = 3.55, df = 5 (P = 0.62), I² = 0%						

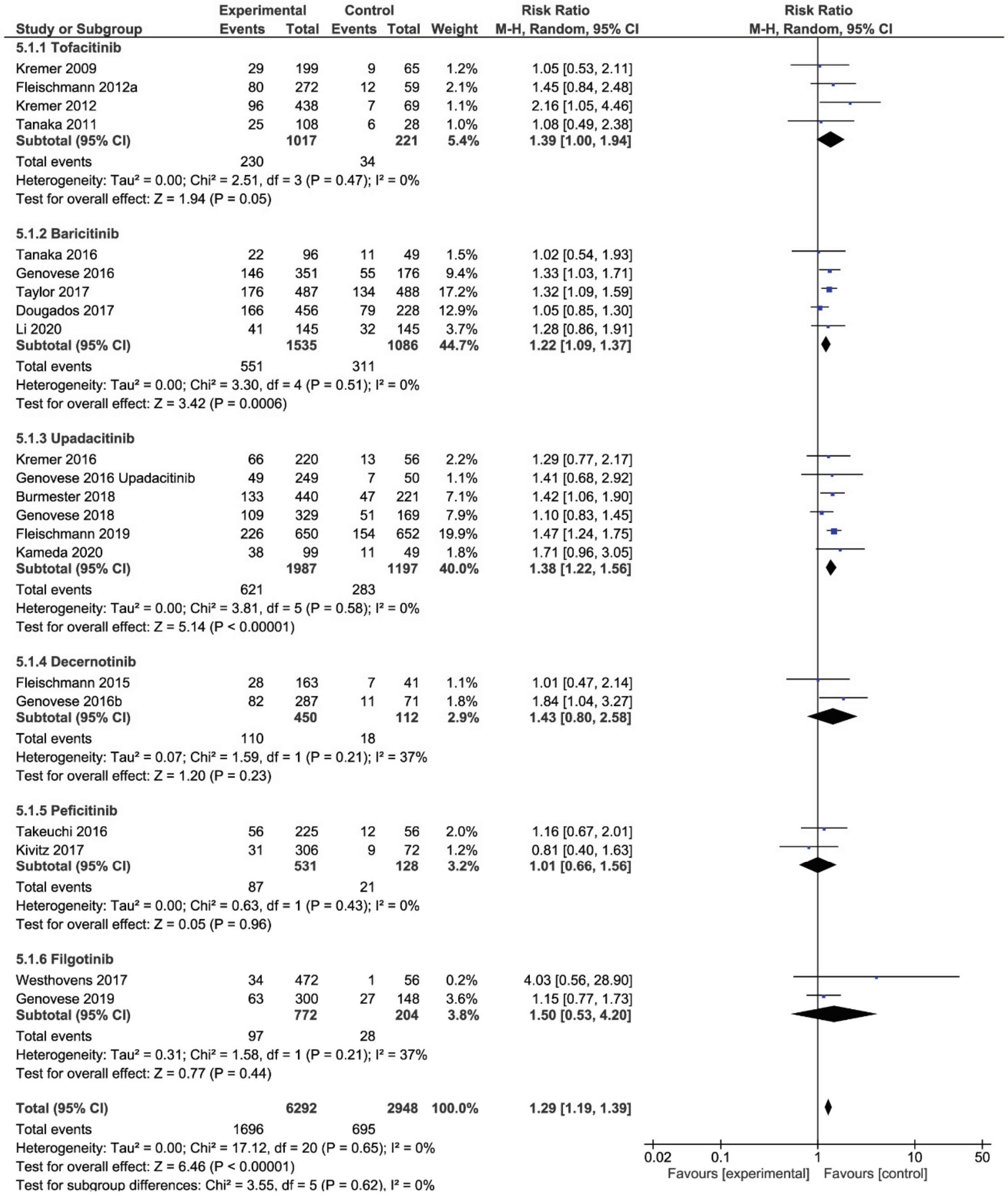

Figure S8 *Forest plot of the effect of JAKinibs on serious infections. Randomized-effects model. RR=Relative Risks.*

Study or Subgroup	Experimental Events	Experimental Total	Control Events	Control Total	Weight	Risk Ratio M-H, Random, 95% CI
**6.1.1 Tofacitinib**						
Vollenhoven 2012	7	405	1	108	2.9%	1.87 [0.23, 15.01]
Fleischmann 2012a	3	272	1	59	2.5%	0.65 [0.07, 6.15]
Fleischmann 2012b	1	488	0	122	1.2%	0.75 [0.03, 18.41]
Kremer 2012	5	438	0	69	1.5%	1.75 [0.10, 31.37]
Heijde 2013	43	637	7	160	20.6%	1.54 [0.71, 3.37]
Burmester 2013	0	267	0	132		Not estimable
Boyle 2015	0	15	0	14		Not estimable
Tanaka 2015	4	265	1	52	2.7%	0.78 [0.09, 6.88]
**Subtotal (95% CI)**		**2787**		**716**	**31.4%**	**1.35 [0.72, 2.55]**
Total events	63		10			
Heterogeneity: Tau² = 0.00; Chi² = 1.02, df = 5 (P = 0.96); I² = 0%						
Test for overall effect: Z = 0.94 (P = 0.35)						
**6.1.2 Baricitinib**						
Keystone 2015	2	203	0	98	1.4%	2.43 [0.12, 50.06]
Tanaka 2016	0	96	0	49		Not estimable
Genovese 2016	10	351	5	176	11.2%	1.00 [0.35, 2.89]
Taylor 2017	5	487	7	488	9.6%	0.72 [0.23, 2.24]
Dougados 2017	6	456	4	228	8.0%	0.75 [0.21, 2.63]
Li 2020	1	145	0	145	1.2%	3.00 [0.12, 73.04]
**Subtotal (95% CI)**		**1738**		**1184**	**31.4%**	**0.91 [0.48, 1.71]**
Total events	24		16			
Heterogeneity: Tau² = 0.00; Chi² = 1.24, df = 4 (P = 0.87); I² = 0%						
Test for overall effect: Z = 0.29 (P = 0.77)						
**6.1.3 Upadacitinib**						
Kremer 2016	0	220	1	56	1.2%	0.09 [0.00, 2.08]
Genovese 2016 Upadacitinib	1	249	0	50	1.2%	0.61 [0.03, 14.81]
Burmester 2018	4	440	1	221	2.6%	2.01 [0.23, 17.87]
Genovese 2018	5	329	0	169	1.5%	5.67 [0.32, 101.87]
Fleischmann 2019	12	650	5	652	11.6%	2.41 [0.85, 6.79]
Kameda 2020	4	99	0	49	1.5%	4.50 [0.25, 81.94]
**Subtotal (95% CI)**		**1987**		**1197**	**19.7%**	**1.92 [0.83, 4.47]**
Total events	26		7			
Heterogeneity: Tau² = 0.05; Chi² = 5.20, df = 5 (P = 0.39); I² = 4%						
Test for overall effect: Z = 1.51 (P = 0.13)						
**6.1.4 Decernotinib**						
Fleischmann 2015	5	163	0	41	1.5%	2.82 [0.16, 49.94]
Genovese 2016b	10	287	1	71	3.0%	2.47 [0.32, 19.01]
**Subtotal (95% CI)**		**450**		**112**	**4.5%**	**2.58 [0.49, 13.63]**
Total events	15		1			
Heterogeneity: Tau² = 0.00; Chi² = 0.01, df = 1 (P = 0.94); I² = 0%						
Test for overall effect: Z = 1.12 (P = 0.26)						
**6.1.5 Peficitinib**						
Genovese 2017	1	238	0	51	1.2%	0.65 [0.03, 15.80]
Kivitz 2017	2	306	0	72	1.4%	1.19 [0.06, 24.50]
Takeuchi 2019	12	348	0	170	1.6%	12.25 [0.73, 205.66]
Tanaka 2019	3	206	0	101	1.4%	3.45 [0.18, 66.15]
**Subtotal (95% CI)**		**1098**		**394**	**5.6%**	**2.63 [0.59, 11.73]**
Total events	18		0			
Heterogeneity: Tau² = 0.00; Chi² = 2.49, df = 3 (P = 0.48); I² = 0%						
Test for overall effect: Z = 1.27 (P = 0.20)						
**6.1.6 Filgotinib**						
Kavanaugh 2017	2	211	0	72	1.4%	1.72 [0.08, 35.45]
Westhovens 2017	5	472	1	56	2.8%	0.59 [0.07, 4.99]
Genovese 2019	2	300	2	148	3.3%	0.49 [0.07, 3.47]
**Subtotal (95% CI)**		**983**		**276**	**7.4%**	**0.67 [0.18, 2.44]**
Total events	9		3			
Heterogeneity: Tau² = 0.00; Chi² = 0.49, df = 2 (P = 0.78); I² = 0%						
Test for overall effect: Z = 0.62 (P = 0.54)						
**Total (95% CI)**		**9043**		**3879**	**100.0%**	**1.30 [0.92, 1.86]**
Total events	155		37			
Heterogeneity: Tau² = 0.00; Chi² = 15.35, df = 25 (P = 0.93); I² = 0%						
Test for overall effect: Z = 1.47 (P = 0.14)						
Test for subgroup differences: Chi² = 4.58, df = 5 (P = 0.47), I² = 0%						

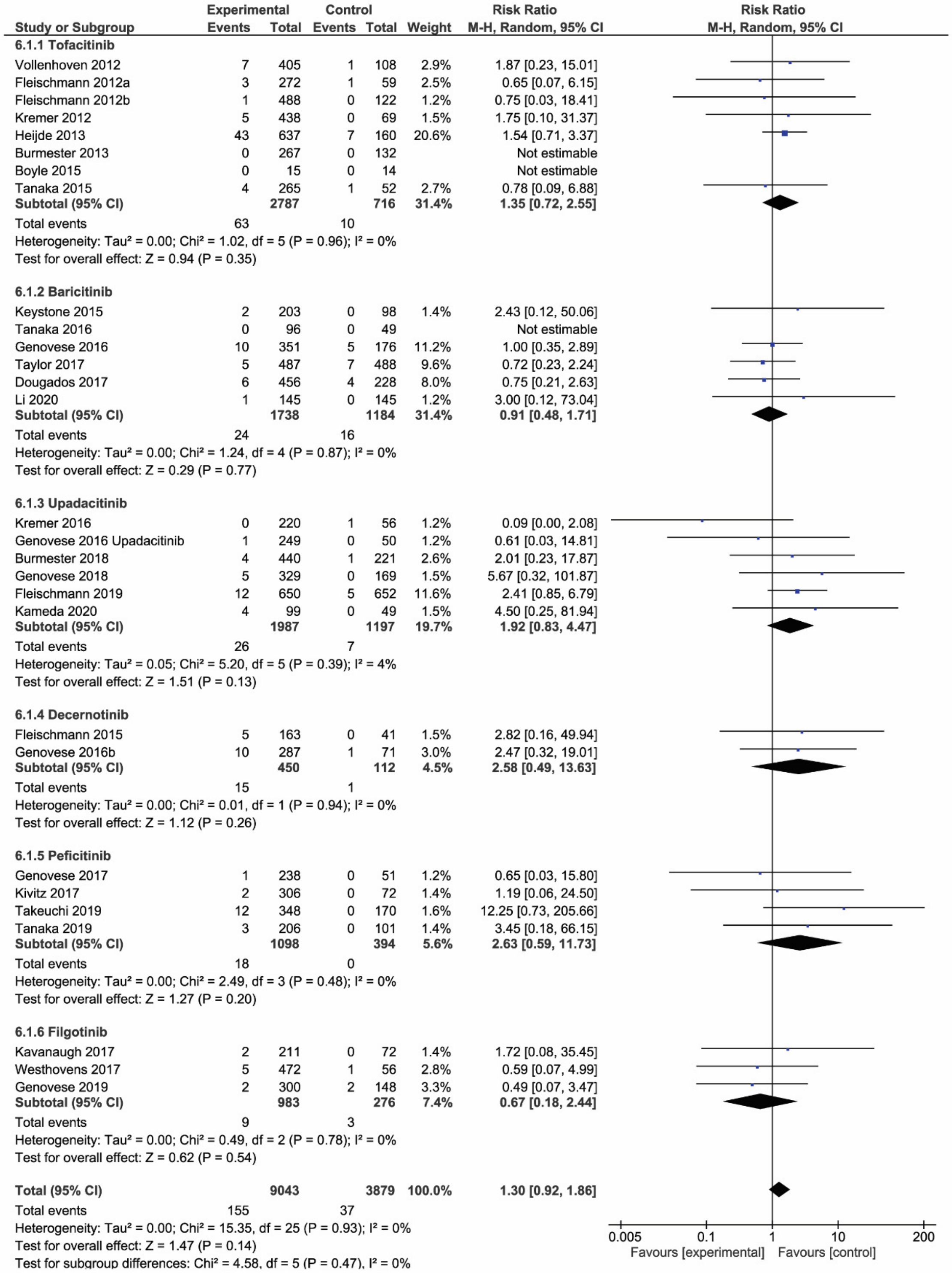

Figure S9 *Forest plot of the effect of JAKinibs on HZ. Randomized-effects model. RR=Relative Risks.*

Study or Subgroup	Experimental Events	Experimental Total	Control Events	Control Total	Weight	Risk Ratio M-H, Random, 95% CI
**7.1.1 Tofacitinib**						
Kremer 2009	1	199	1	65	1.9%	0.33 [0.02, 5.15]
Vollenhoven 2012	6	405	0	108	1.7%	3.49 [0.20, 61.47]
Heijde 2013	56	637	11	160	36.7%	1.28 [0.69, 2.38]
Tanaka 2015	5	265	0	52	1.7%	2.19 [0.12, 39.04]
**Subtotal (95% CI)**		**1506**		**385**	**42.0%**	**1.28 [0.72, 2.29]**
Total events	68		12			
Heterogeneity: Tau² = 0.00; Chi² = 1.56, df = 3 (P = 0.67); I² = 0%						
Test for overall effect: Z = 0.84 (P = 0.40)						
**7.1.2 Baricitinib**						
Keystone 2015	0	203	0	98		Not estimable
Tanaka 2016	0	96	0	49		Not estimable
Genovese 2016	9	351	2	176	6.1%	2.26 [0.49, 10.33]
Taylor 2017	7	487	2	488	5.8%	3.51 [0.73, 16.80]
Dougados 2017	7	456	0	228	1.7%	7.52 [0.43, 131.03]
Li 2020	1	145	0	145	1.4%	3.00 [0.12, 73.04]
**Subtotal (95% CI)**		**1738**		**1184**	**15.1%**	**3.15 [1.19, 8.33]**
Total events	24		4			
Heterogeneity: Tau² = 0.00; Chi² = 0.57, df = 3 (P = 0.90); I² = 0%						
Test for overall effect: Z = 2.32 (P = 0.02)						
**7.1.3 Upadacitinib**						
Kremer 2016	3	220	2	56	4.6%	0.38 [0.07, 2.23]
Genovese 2016 Upadacitinib	3	249	0	50	1.6%	1.43 [0.07, 27.22]
Burmester 2018	3	440	1	221	2.8%	1.51 [0.16, 14.40]
Genovese 2018	5	329	1	169	3.1%	2.57 [0.30, 21.81]
Fleischmann 2019	5	650	3	652	7.0%	1.67 [0.40, 6.97]
Kameda 2020	3	99	1	49	2.8%	1.48 [0.16, 13.91]
**Subtotal (95% CI)**		**1987**		**1197**	**21.9%**	**1.25 [0.56, 2.81]**
Total events	22		8			
Heterogeneity: Tau² = 0.00; Chi² = 2.41, df = 5 (P = 0.79); I² = 0%						
Test for overall effect: Z = 0.55 (P = 0.58)						
**7.1.4 Decernotinib**						
Fleischmann 2015	3	163	0	41	1.6%	1.79 [0.09, 34.04]
**Subtotal (95% CI)**		**163**		**41**	**1.6%**	**1.79 [0.09, 34.04]**
Total events	3		0			
Heterogeneity: Not applicable						
Test for overall effect: Z = 0.39 (P = 0.70)						
**7.1.5 Peficitinib**						
Genovese 2017	0	238	1	51	1.4%	0.07 [0.00, 1.76]
Kivitz 2017	3	306	0	72	1.6%	1.66 [0.09, 31.87]
Takeuchi 2019	19	348	2	170	6.8%	4.64 [1.09, 19.69]
Tanaka 2019	9	206	0	101	1.8%	9.36 [0.55, 159.27]
Takeuchi 2016	4	225	0	56	1.7%	2.27 [0.12, 41.55]
**Subtotal (95% CI)**		**1323**		**450**	**13.3%**	**2.13 [0.51, 8.92]**
Total events	35		3			
Heterogeneity: Tau² = 0.98; Chi² = 6.39, df = 4 (P = 0.17); I² = 37%						
Test for overall effect: Z = 1.04 (P = 0.30)						
**7.1.6 Filgotinib**						
Kavanaugh 2017	1	211	0	72	1.4%	1.03 [0.04, 25.08]
Westhovens 2017	4	472	1	56	3.0%	0.47 [0.05, 4.17]
Genovese 2019	3	300	0	148	1.6%	3.47 [0.18, 66.65]
**Subtotal (95% CI)**		**983**		**276**	**6.0%**	**0.97 [0.21, 4.51]**
Total events	8		1			
Heterogeneity: Tau² = 0.00; Chi² = 1.22, df = 2 (P = 0.54); I² = 0%						
Test for overall effect: Z = 0.04 (P = 0.97)						
**Total (95% CI)**		**7700**		**3533**	**100.0%**	**1.59 [1.09, 2.32]**
Total events	160		28			
Heterogeneity: Tau² = 0.00; Chi² = 16.57, df = 22 (P = 0.79); I² = 0%						
Test for overall effect: Z = 2.41 (P = 0.02)						
Test for subgroup differences: Chi² = 3.27, df = 5 (P = 0.66), I² = 0%						

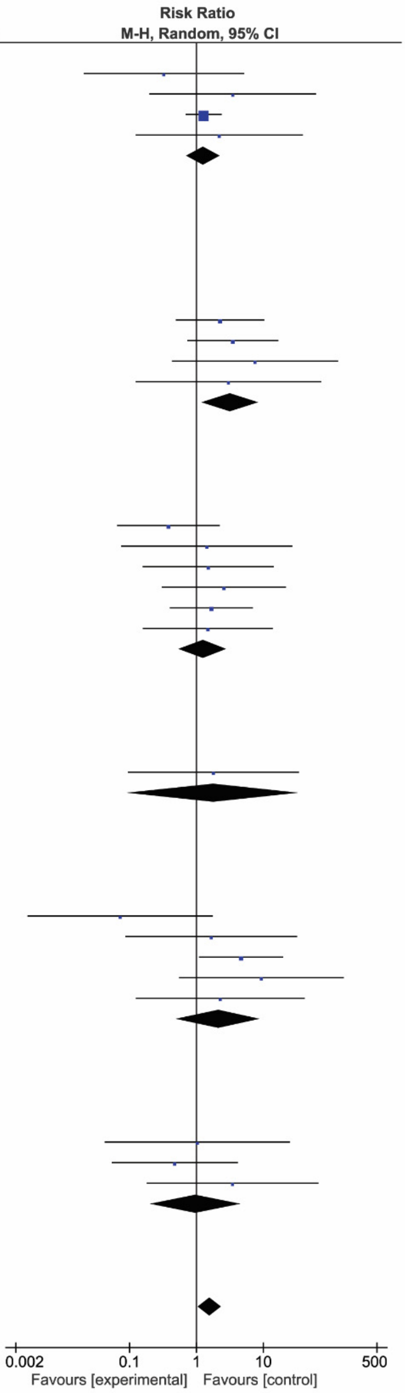

Figure S10 *Forest plot of the effect of JAKinibs on upper respiratory infection. Randomized-effects model. RR=Relative Risks.*

Study or Subgroup	Experimental Events	Experimental Total	Control Events	Control Total	Weight	Risk Ratio M-H, Random, 95% CI
**8.1.1 Tofacitinib**						
Kremer 2009	4	199	4	65	3.6%	0.33 [0.08, 1.27]
Vollenhoven 2012	16	405	1	108	1.6%	4.27 [0.57, 31.81]
Fleischmann 2012a	10	272	2	59	3.0%	1.08 [0.24, 4.82]
Fleischmann 2012b	19	488	6	122	8.2%	0.79 [0.32, 1.94]
Kremer 2012	19	438	2	69	3.2%	1.50 [0.36, 6.28]
Burmester 2013	7	267	4	132	4.5%	0.87 [0.26, 2.90]
Kremer 2013	87	779	7	159	11.7%	2.54 [1.20, 5.38]
Tanaka 2015	4	265	0	52	0.8%	1.79 [0.10, 32.81]
**Subtotal (95% CI)**		**3113**		**766**	**36.4%**	**1.20 [0.69, 2.10]**
Total events	166		26			
Heterogeneity: Tau² = 0.20; Chi² = 10.50, df = 7 (P = 0.16); I² = 33%						
Test for overall effect: Z = 0.64 (P = 0.52)						
**8.1.2 Baricitinib**						
Genovese 2016	26	351	8	176	11.0%	1.63 [0.75, 3.52]
Dougados 2017	38	456	18	228	22.7%	1.06 [0.62, 1.81]
**Subtotal (95% CI)**		**807**		**404**	**33.8%**	**1.22 [0.78, 1.89]**
Total events	64		26			
Heterogeneity: Tau² = 0.00; Chi² = 0.82, df = 1 (P = 0.36); I² = 0%						
Test for overall effect: Z = 0.87 (P = 0.38)						
**8.1.3 Upadacitinib**						
Burmester 2018	24	440	9	221	11.7%	1.34 [0.63, 2.83]
**Subtotal (95% CI)**		**440**		**221**	**11.7%**	**1.34 [0.63, 2.83]**
Total events	24		9			
Heterogeneity: Not applicable						
Test for overall effect: Z = 0.76 (P = 0.44)						
**8.1.4 Decernotinib**						
Genovese 2016b	10	287	2	71	2.9%	1.24 [0.28, 5.52]
**Subtotal (95% CI)**		**287**		**71**	**2.9%**	**1.24 [0.28, 5.52]**
Total events	10		2			
Heterogeneity: Not applicable						
Test for overall effect: Z = 0.28 (P = 0.78)						
**8.1.5 Peficitinib**						
Genovese 2017	13	238	2	51	3.1%	1.39 [0.32, 5.98]
Kivitz 2017	29	306	4	72	6.4%	1.71 [0.62, 4.70]
**Subtotal (95% CI)**		**544**		**123**	**9.5%**	**1.60 [0.69, 3.67]**
Total events	42		6			
Heterogeneity: Tau² = 0.00; Chi² = 0.05, df = 1 (P = 0.82); I² = 0%						
Test for overall effect: Z = 1.10 (P = 0.27)						
**8.1.6 Filgotinib**						
Genovese 2019	9	300	5	148	5.7%	0.89 [0.30, 2.60]
**Subtotal (95% CI)**		**300**		**148**	**5.7%**	**0.89 [0.30, 2.60]**
Total events	9		5			
Heterogeneity: Not applicable						
Test for overall effect: Z = 0.22 (P = 0.83)						
**Total (95% CI)**		**5491**		**1733**	**100.0%**	**1.26 [0.97, 1.63]**
Total events	315		74			
Heterogeneity: Tau² = 0.00; Chi² = 11.98, df = 14 (P = 0.61); I² = 0%						
Test for overall effect: Z = 1.76 (P = 0.08)						
Test for subgroup differences: Chi² = 0.79, df = 5 (P = 0.98), I² = 0%						

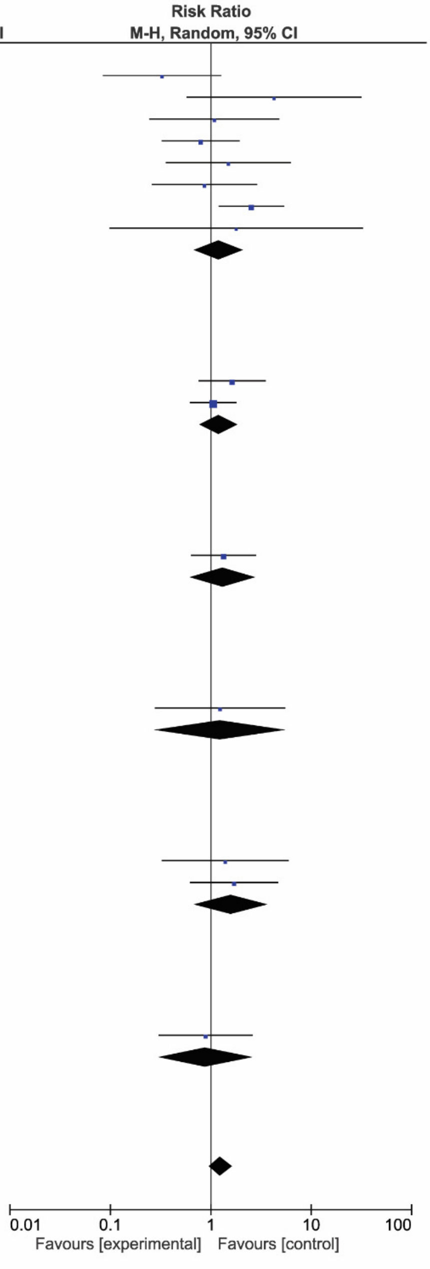

Figure S11 *Forest plot of the effect of JAKinibs on thromboembolic events. Randomized-effects model. RR=Relative Risks.*

Study or Subgroup	Experimental Events	Experimental Total	Control Events	Control Total	Weight	Risk Ratio M-H, Random, 95% CI
**9.1.1 Tofacitinib**						
Fleischmann 2012b	0	488	2	122	10.9%	0.05 [0.00, 1.04]
Vollenhoven 2012	1	405	0	108	9.8%	0.81 [0.03, 19.63]
**Subtotal (95% CI)**		**893**		**230**	**20.8%**	**0.19 [0.01, 2.91]**
Total events	1		2			
Heterogeneity: Tau² = 1.33; Chi² = 1.53, df = 1 (P = 0.22); I² = 35%						
Test for overall effect: Z = 1.19 (P = 0.23)						
**9.1.2 Baricitinib**						
Genovese 2016	1	456	0	228	9.8%	1.50 [0.06, 36.76]
Dougados 2017	3	351	0	176	11.5%	3.52 [0.18, 67.77]
**Subtotal (95% CI)**		**807**		**404**	**21.3%**	**2.38 [0.27, 20.84]**
Total events	4		0			
Heterogeneity: Tau² = 0.00; Chi² = 0.15, df = 1 (P = 0.70); I² = 0%						
Test for overall effect: Z = 0.78 (P = 0.43)						
**9.1.3 Upadacitinib**						
Kremer 2016	2	220	0	56	11.0%	1.29 [0.06, 26.49]
Burmester 2018	0	440	0	221		Not estimable
Genovese 2018	1	329	0	169	9.8%	1.55 [0.06, 37.73]
Fleischmann 2019	2	650	1	652	17.4%	2.01 [0.18, 22.07]
Kameda 2020	0	99	0	49		Not estimable
**Subtotal (95% CI)**		**1738**		**1147**	**38.2%**	**1.65 [0.33, 8.35]**
Total events	5		1			
Heterogeneity: Tau² = 0.00; Chi² = 0.05, df = 2 (P = 0.97); I² = 0%						
Test for overall effect: Z = 0.61 (P = 0.54)						
**9.1.4 Decernotinib**						
Fleischmann 2015	1	163	0	41	9.9%	0.77 [0.03, 18.52]
**Subtotal (95% CI)**		**163**		**41**	**9.9%**	**0.77 [0.03, 18.52]**
Total events	1		0			
Heterogeneity: Not applicable						
Test for overall effect: Z = 0.16 (P = 0.87)						
**9.1.5 Peficitinib**						
Takeuchi 2019	0	348	0	170		Not estimable
Tanaka 2019	0	206	0	101		Not estimable
**Subtotal (95% CI)**		**554**		**271**		**Not estimable**
Total events	0		0			
Heterogeneity: Not applicable						
Test for overall effect: Not applicable						
**9.1.6 Filgotinib**						
Genovese 2019	1	300	0	148	9.8%	1.49 [0.06, 36.24]
**Subtotal (95% CI)**		**300**		**148**	**9.8%**	**1.49 [0.06, 36.24]**
Total events	1		0			
Heterogeneity: Not applicable						
Test for overall effect: Z = 0.24 (P = 0.81)						
**Total (95% CI)**		**4455**		**2241**	**100.0%**	**1.04 [0.38, 2.84]**
Total events	12		3			
Heterogeneity: Tau² = 0.00; Chi² = 5.03, df = 8 (P = 0.75); I² = 0%						
Test for overall effect: Z = 0.08 (P = 0.94)						
Test for subgroup differences: Chi² = 2.37, df = 4 (P = 0.67), I² = 0%						

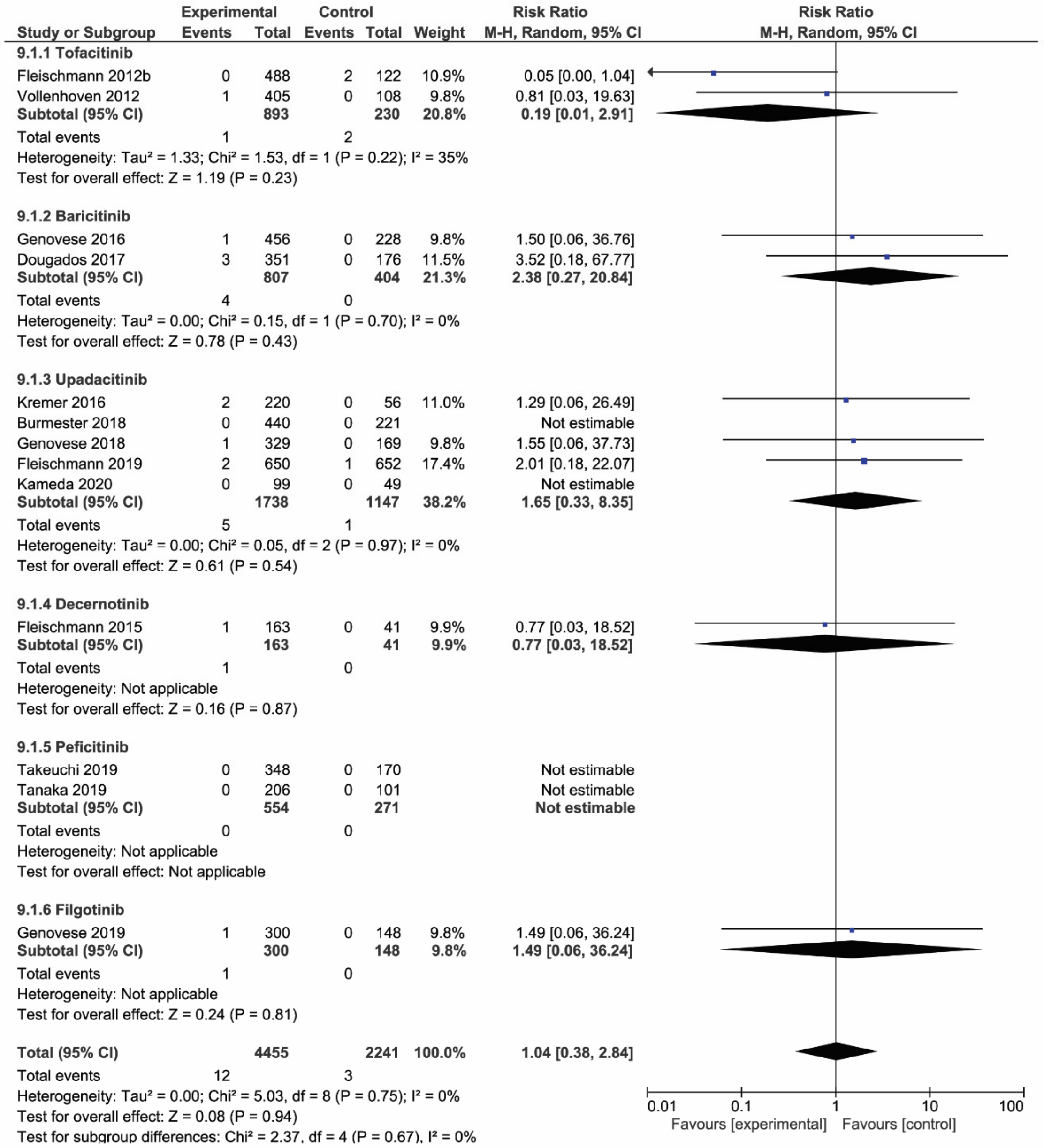

Figure S12 *Forest plot of the effect of JAKinibs on MACE (major adverse cardiovascular events). Randomized-effects model. RR=Relative Risks.*

Study or Subgroup	Experimental Events	Experimental Total	Control Events	Control Total	Weight	Risk Ratio M-H, Random, 95% CI
**10.1.1 Tofacitinib**						
Fleischmann 2012b	0	488	0	122		Not estimable
Kremer 2013	3	633	0	159	7.9%	1.77 [0.09, 34.03]
Heijde 2013	6	637	0	160	8.3%	3.28 [0.19, 57.93]
**Subtotal (95% CI)**		**1758**		**441**	**16.2%**	**2.43 [0.31, 19.07]**
Total events	9		0			
Heterogeneity: Tau² = 0.00; Chi² = 0.09, df = 1 (P = 0.77); I² = 0%						
Test for overall effect: Z = 0.84 (P = 0.40)						
**10.1.2 Baricitinib**						
Keystone 2015	0	203	1	98	6.7%	0.16 [0.01, 3.94]
Genovese 2016	2	351	0	176	7.5%	2.51 [0.12, 52.09]
Taylor 2017	1	487	0	488	6.7%	3.01 [0.12, 73.61]
Dougados 2017	0	456	2	228	7.5%	0.10 [0.00, 2.08]
Li 2020	0	145	0	145		Not estimable
**Subtotal (95% CI)**		**1642**		**1135**	**28.4%**	**0.59 [0.10, 3.40]**
Total events	3		3			
Heterogeneity: Tau² = 0.69; Chi² = 3.82, df = 3 (P = 0.28); I² = 21%						
Test for overall effect: Z = 0.59 (P = 0.55)						
**10.1.3 Upadacitinib**						
Kremer 2016	1	220	0	56	6.8%	0.77 [0.03, 18.74]
Genovese 2016 Upadacitinib	1	249	0	50	6.8%	0.61 [0.03, 14.81]
Genovese 2018	1	329	0	169	6.7%	1.55 [0.06, 37.73]
Fleischmann 2019	3	651	2	651	21.6%	1.50 [0.25, 8.95]
Kameda 2020	0	99	0	49		Not estimable
**Subtotal (95% CI)**		**1548**		**975**	**41.8%**	**1.17 [0.32, 4.22]**
Total events	6		2			
Heterogeneity: Tau² = 0.00; Chi² = 0.33, df = 3 (P = 0.95); I² = 0%						
Test for overall effect: Z = 0.24 (P = 0.81)						
**10.1.4 Decernotinib**						
Fleischmann 2015	1	163	0	41	6.8%	0.77 [0.03, 18.52]
Genovese 2016b	1	287	0	71	6.8%	0.75 [0.03, 18.22]
**Subtotal (95% CI)**		**450**		**112**	**13.5%**	**0.76 [0.08, 7.22]**
Total events	2		0			
Heterogeneity: Tau² = 0.00; Chi² = 0.00, df = 1 (P = 0.99); I² = 0%						
Test for overall effect: Z = 0.24 (P = 0.81)						
**10.1.5 Peficitinib**						
Kivitz 2017	0	306	0	72		Not estimable
**Subtotal (95% CI)**		**306**		**72**		**Not estimable**
Total events	0		0			
Heterogeneity: Not applicable						
Test for overall effect: Not applicable						
**Total (95% CI)**		**5704**		**2735**	**100.0%**	**1.02 [0.45, 2.34]**
Total events	20		5			
Heterogeneity: Tau² = 0.00; Chi² = 5.51, df = 11 (P = 0.90); I² = 0%						
Test for overall effect: Z = 0.05 (P = 0.96)						
Test for subgroup differences: Chi² = 1.17, df = 3 (P = 0.76), I² = 0%						

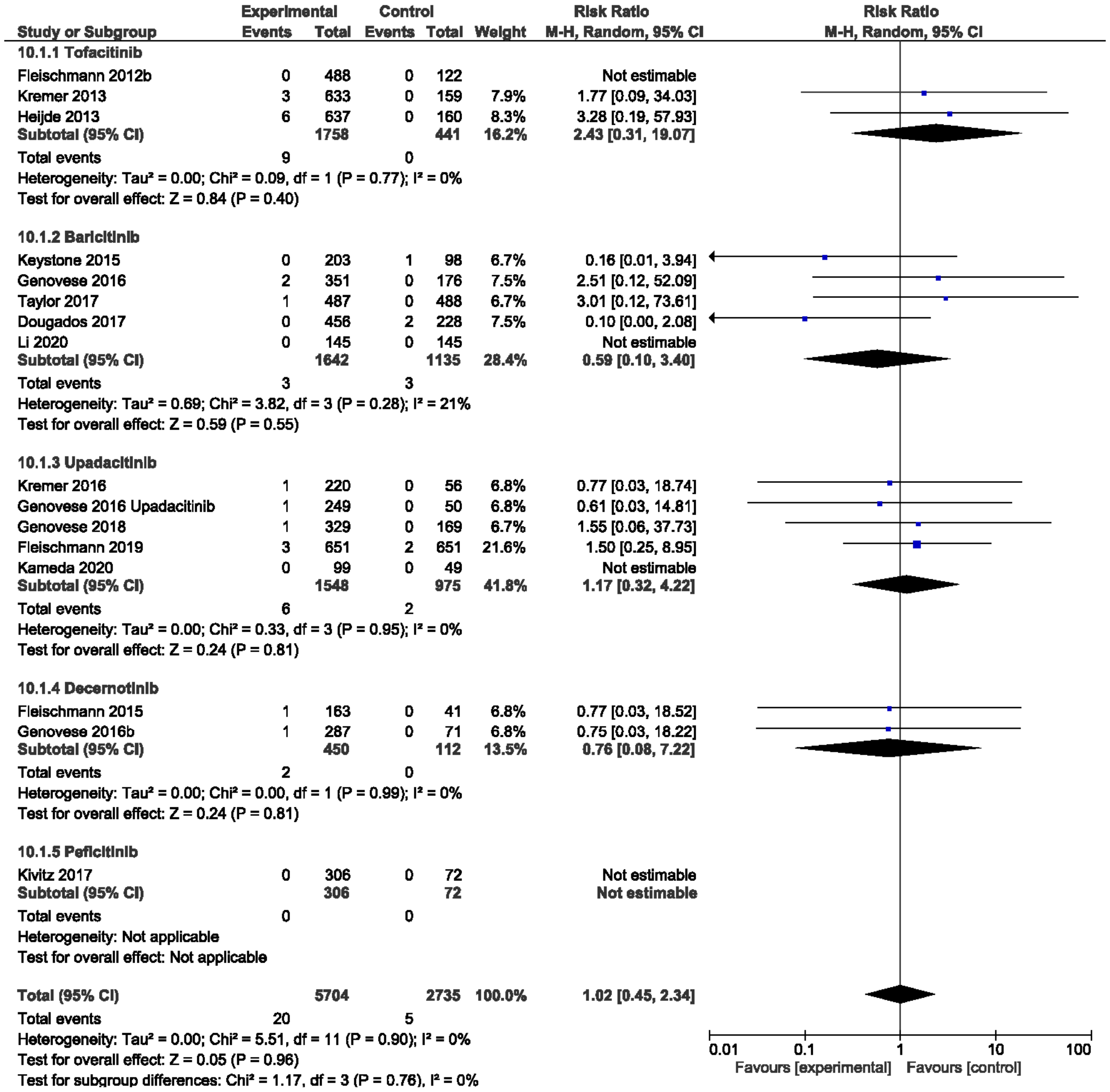

Figure S13 *Forest plot of the effect of JAKinibs on neoplasms. Randomized-effects model. RR=Relative Risks.*

Study or Subgroup	Experimental Events	Experimental Total	Control Events	Control Total	Weight	Risk Ratio M-H, Random, 95% CI
**11.1.1 Tofacitinib**						
Heijde 2013	9	321	0	160	8.6%	9.50 [0.56, 162.20]
**Subtotal (95% CI)**		**321**		**160**	**8.6%**	**9.50 [0.56, 162.20]**
Total events	9		0			
Heterogeneity: Not applicable						
Test for overall effect: Z = 1.56 (P = 0.12)						
**11.1.2 Baricitinib**						
Tanaka 2016	0	96	0	49		Not estimable
Genovese 2016	2	351	0	176	7.5%	2.51 [0.12, 52.09]
Taylor 2017	2	487	3	488	21.6%	0.67 [0.11, 3.98]
Dougados 2017	1	456	0	228	6.7%	1.50 [0.06, 36.76]
Li 2020	0	145	0	145		Not estimable
**Subtotal (95% CI)**		**1535**		**1086**	**35.9%**	**1.03 [0.26, 4.10]**
Total events	5		3			
Heterogeneity: Tau² = 0.00; Chi² = 0.62, df = 2 (P = 0.73); I² = 0%						
Test for overall effect: Z = 0.04 (P = 0.97)						
**11.1.3 Upadacitinib**						
Kremer 2016	1	220	0	56	6.8%	0.77 [0.03, 18.74]
Genovese 2016 Upadacitinib	1	249	0	50	6.8%	0.61 [0.03, 14.81]
Genovese 2018	3	329	0	169	7.9%	3.61 [0.19, 69.41]
Burmester 2018	1	440	0	221	6.7%	1.51 [0.06, 36.92]
Fleischmann 2019	2	650	1	652	12.0%	2.01 [0.18, 22.07]
Kameda 2020	0	99	0	49		Not estimable
**Subtotal (95% CI)**		**1987**		**1197**	**40.2%**	**1.50 [0.40, 5.54]**
Total events	8		1			
Heterogeneity: Tau² = 0.00; Chi² = 0.89, df = 4 (P = 0.93); I² = 0%						
Test for overall effect: Z = 0.60 (P = 0.55)						
**11.1.4 Peficitinib**						
Takeuchi 2016	0	225	0	56		Not estimable
Genovese 2017	0	238	0	51		Not estimable
Kivitz 2017	0	306	0	72		Not estimable
Takeuchi 2019	3	348	0	170	7.9%	3.43 [0.18, 66.03]
Tanaka 2019	2	206	0	101	7.5%	2.46 [0.12, 50.85]
**Subtotal (95% CI)**		**1323**		**450**	**15.4%**	**2.92 [0.35, 24.20]**
Total events	5		0			
Heterogeneity: Tau² = 0.00; Chi² = 0.02, df = 1 (P = 0.88); I² = 0%						
Test for overall effect: Z = 0.99 (P = 0.32)						
**11.1.5 Filgotinib**						
Kavanaugh 2017	0	211	0	72		Not estimable
Westhovens 2017	0	508	0	86		Not estimable
**Subtotal (95% CI)**		**719**		**158**		**Not estimable**
Total events	0		0			
Heterogeneity: Not applicable						
Test for overall effect: Not applicable						
**Total (95% CI)**		**5885**		**3051**	**100.0%**	**1.70 [0.74, 3.89]**
Total events	27		4			
Heterogeneity: Tau² = 0.00; Chi² = 3.95, df = 10 (P = 0.95); I² = 0%						
Test for overall effect: Z = 1.25 (P = 0.21)						
Test for subgroup differences: Chi² = 2.21, df = 3 (P = 0.53), I² = 0%						

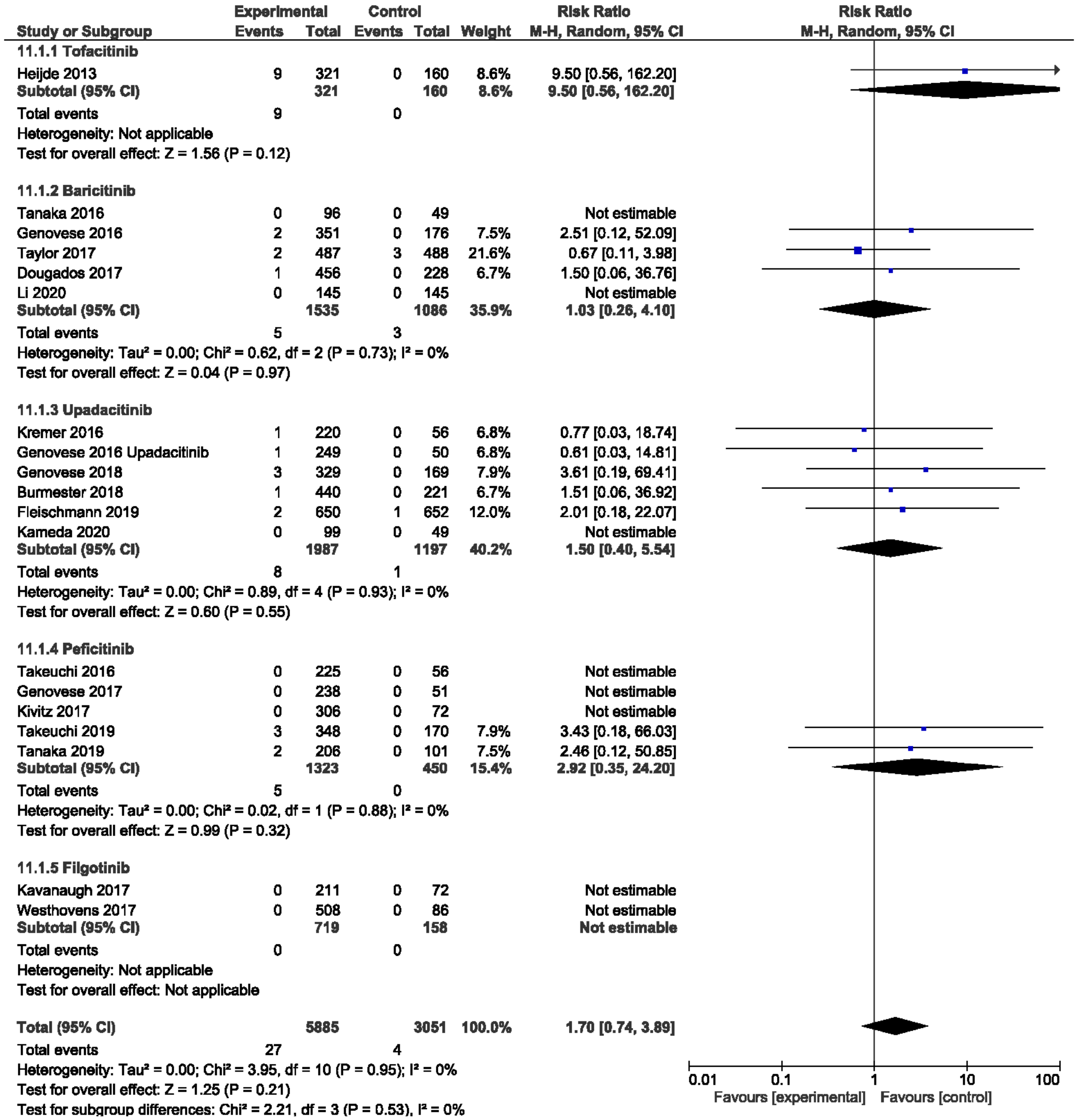

Table S1 Sensitivity analyses of ACR20 and ACR50 in RCTsStratification	ACR20				ACR50			
	No. of Patients (Studies)	RR(95% CI)	P Value	I^2, %	No. of Patients (Studies)	RR (95% CI)	P Value	I^2, %
**Therapy**								
monotherapy	12326(29)	1.94(1.76-2.14)	<0.001	66	12041 (29)	3.10(2.64-3.64)	<0.001	58
Combination with MTX only	7963(19)	2.11(1.86-2.39)	<0.001	66	8462(20)	2.86(2.231-3.54)	<0.001	69
Combination with DMARDs	8943(24)	2.11(1.86-2.39)	<0.001	64	8943(24)	3.51(2.70-4.56)	<0.001	74
**Trials**								
Phase II	8662(15)	2.08(1.88-2.31)	<0.001	63	8243(15)	2.97(2.42-3.64)	<0.001	73
Phase III	5954(21)	2.03(1.72-2.39)	<0.001	67	5954(21)	3.47(2.59-4.64)	<0.001	58
**No. of subjects**								
<100	14417(32)	0.63(0.28-1.45)	<0.001	67	13969(31)	3.08(2.61-3.65)	<0.001	69
≥100	199(4)	2.12(1.09-4.10)	0.14	45	199(4)	4.49(1.58-12.80)	0.88	0
**Follow-up**								
≤12 week	7395(13)	2.11(1.81-2.46)	<0.001	74	6947(12)	2.93(2.24-3.83)	<0.001	77
>12 week	7221(23)	1.98(1.76-2.23)	<0.001	67	7221(23)	3.27(2.64-3.97)	0.003	51
**Patients**								
MTX-IR	7668(20)	2.02(1.81-2.26)	0.001	56	6702(17)	3.00(2.34-3.83)	<0.001	66
DMARD-IR	9168(23)	1.99(1.77-2.24)	<0.001	65	8323 (19)	3.11(2.55-3.78)	<0.001	62
**Studies**								
Non-multicenter	13035(29)	1.93(1.76-2.11)	<0.001	62	12587(28)	2.90(2.44-3.44)	<0.001	67
One-study-out method	…	…	From 1.99 (1.82-2.18) to 2.07 (1.89-2.26)	…		…	From 3.03(2.58-3.57) to 3.18 (2.68-3.78)	

bDMARD = biologic disease-modifying antirheumatic drug; csDMARD = conventional synthetic disease-modifying antirheumatic drug such as methotrexate; DMARD = disease-modifying antirheumatic drug; IR = inadequate response; MTX =methotrexate.

Table S2. Search strategies

Source:medline

Searched on: May 5, 2021

Results: 568

Search	***Query***	***Results***
1	*(tofacitinib or cp 690 550 or cp 690550 or cp690 550 or cp690550 or tasocitinib or tofacitinib or xeljanz).mp.*	*4887*
2	*(baricitinib or incb 028050or incb 28050 or incb028050 or incb28050 or ly 3009104 or ly3009104 or olumiant).mp.*	*1095*
3	*(upadcitinib or ABT-494 or Rinvoq).mp.*	*87*
4	*(decernotinib or VX-509).mp.*	*126*
5	*(peficitinib or ASP015K or Smyraf).mp.*	*195*
6	*(filgotinib or GS-6034 or GLPG0634 or GLPG0634).mp.*	*538*
7	*1 or 2 or 3 or 4 or 5 or 6*	*6863*
8	*(rheumatoid arthritis).mp.*	*200385*
9	*exp arthritis, rheumatoid*	*193438*
10	*7 and 9*	*2734*

11	*10 and exp evidence-based medicine/*	*649*
12	*exp controlled clinical trial/*	*767599*
13	*9 and 12*	*373*
14	*11 or 13*	*486*
15	*remove duplicates from 14*	*568*

Source: Embase
Searched on: May 5, 2021
Results: 779

Search	***Query***	***Results***
1	*(tofacitinib or cp 690 550 or cp 690550 or cp690 550 or cp690550 or tasocitinib or tofacitinib or xeljanz).mp.*	*6202*
2	*(baricitinib or incb 028050or incb 28050 or incb028050 or incb28050 or ly 3009104 or ly3009104 or olumiant).mp.*	*2185*
3	*(upadcitinib or ABT-494 or Rinvoq).mp.*	*137*
4	*(decernotinib or VX-509).mp.*	*145*
5	*(peficitinib or ASP015K or Smyraf).mp.*	*217*
6	*(filgotinib or GS-6034 or GLPG0634 or GLPG0634).mp.*	*633*
7	*1 or 2 or 3 or 4 or 5 or 6*	*8016*
8	*(rheumatoid adj2 arthritis).mp.*	*220123*
9	*exp arthritis, rheumatoid/ or 4.*	*223800*
10	*7 and 9*	*3644*
11	*10 and exp evidence-based medicine/*	*732*
12	*exp controlled clinical trial/*	*852320*
13	*9 and 12*	*463*
14	*11 or 13*	*791*
15	*remove duplicates from 14*	*779*

Source: Cochrane databases
Searched on: May 5, 2021
Results: 792

Search	***Query***	***Results***
1	*(tofacitinib or cp 690 550 or cp 690550 or cp690 550 or cp690550 or tasocitinib or tofacitinib or xeljanz) :ti,ab,kw.*	*5202*
2	*(baricitinib or incb 028050or incb 28050 or incb028050 or incb28050 or ly 3009104 or ly3009104 or olumiant) :ti,ab,kw.*	*1755*
3	*(upadcitinib or ABT-494 or Rinvoq):ti,ab,kw*	*128*
4	*(decernotinib or VX-509):ti,ab,kw.*	*138*
5	*(peficitinib or ASP015K or Smyraf) :ti,ab,kw*	*186*
6	*(filgotinib or GS-6034 or GLPG0634 or GLPG0634) :ti,ab,kw*	*593*
7	*1 or 2 or 3 or 4 or 5 or 6*	*7826*
8	*MeSH descriptor: [rheumatoid arthritis] explode all trees*	*200763*
9	*7 and 8*	*206280*

10	*MeSH descriptor: [controlled clinical trial] explode all trees*	*742328*
11	*9 or 10*	*2984*
12	*remove duplicates from 11*	*792*

PRISMA 2009 Checklist

Section/topic	#	Checklist item	Reported on page #
**TITLE**			Page 1
Title	1	Identify the report as a systematic review, meta-analysis, or both.	
**ABSTRACT**			
Structured summary	2	Provide a structured summary including, as applicable: background; objectives; data sources; study eligibility criteria, participants, and interventions; study appraisal and synthesis methods; results; limitations; conclusions and implications of key findings; systematic review registration number.	Page 2-3
**INTRODUCTION**			
Rationale	3	Describe the rationale for the review in the context of what is already known.	Page 4-5
Objectives	4	Provide an explicit statement of questions being addressed with reference to participants, interventions, comparisons, outcomes, and study design (PICOS).	Page 4-5
**METHODS**			
Protocol and registration	5	Indicate if a review protocol exists, if and where it can be accessed (e.g., Web address), and, if available, provide registration information including registration number.	no
Eligibility criteria	6	Specify study characteristics (e.g., PICOS, length of follow-up) and report characteristics (e.g., years considered, language, publication status) used as criteria for eligibility, giving rationale.	Page 6
Information sources	7	Describe all information sources (e.g., databases with dates of coverage, contact with study authors to identify additional studies) in the search and date last searched.	Page 5
Search	8	Present full electronic search strategy for at least one database, including any limits used, such that it could be repeated.	Page 5
Study selection	9	State the process for selecting studies (i.e., screening, eligibility, included in systematic review, and, if applicable, included in the meta-analysis).	Page 6
Data collection process	10	Describe method of data extraction from reports (e.g., piloted forms, independently, in duplicate) and any processes for obtaining and confirming data from investigators.	Page 6
Data items	11	List and define all variables for which data were sought (e.g., PICOS, funding sources) and any assumptions and simplifications made.	Page 6-7

Risk of bias in individual studies	12	*Describe methods used for assessing risk of bias of individual studies (including specification of whether this was done at the study or outcome level), and how this information is to be used in any data synthesis.*	Page 7
Summary measures	13	*State the principal summary measures (e.g., risk ratio, difference in means).*	Page 7
Synthesis of results	14	*Describe the methods of handling data and combining results of studies, if done, including measures of consistency (e.g., I^2) for each meta-analysis.*	Page 7

From: Moher D, Liberati A, Tetzlaff J, Altman DG, The PRISMA Group (2009). Preferred Reporting Items for Systematic Reviews and Meta-Analyses: The PRISMA Statement. PLoS Med 6(7): e1000097. doi:10.1371/journal.pmed1000097

For more information, visit: ***www.prisma-statement.org****.*
